# Reprogramming the unfolded protein response for replication by porcine reproductive and respiratory syndrome virus

**DOI:** 10.1371/journal.ppat.1008169

**Published:** 2019-11-18

**Authors:** Peng Gao, Yue Chai, Jiangwei Song, Teng Liu, Peng Chen, Lei Zhou, Xinna Ge, Xin Guo, Jun Han, Hanchun Yang

**Affiliations:** Key Laboratory of Animal Epidemiology of the Ministry of Agriculture and Rural Affairs, China Agricultural University College of Veterinary Medicine, Beijing, People’s Republic of China; University of Iowa, UNITED STATES

## Abstract

The unfolded protein response (UPR) in the endoplasmic reticulum (ER) constitutes a critical component of host innate immunity against microbial infections. In this report, we show that porcine reproductive and respiratory syndrome virus (PRRSV) utilizes the UPR machinery for its own benefit. We provide evidence that the virus targets the UPR central regulator GRP78 for proteasomal degradation via a mechanism that requires viral glycoprotein GP2a, while both IRE1-XBP1s and PERK-eIF2α-ATF4 signaling branches of the UPR are turned on at early stage of infection. The activated effector XBP1s was found to enter the nucleus, but ATF4 was unexpectedly diverted to cytoplasmic viral replication complexes by means of nonstructural proteins nsp2/3 to promote viral RNA synthesis. RNAi knockdown of either ATF4 or XBP1s dramatically attenuated virus titers, while overexpression caused increases. These observations reveal attractive host targets (e.g., ATF4 and XBP1s) for antiviral drugs and have implications in vaccine development.

## Author summary

Porcine reproductive and respiratory syndrome virus (PRRSV) poses a major threat to the worldwide swine industry, but no effective vaccines or antiviral drugs are available. A better understanding of the pathogen-host interactions that support PRRSV replication is essential for understanding viral pathogenesis and the development of preventive measures. Here we report that PRRSV utilizes unconventional strategies to reprogram the unfolded protein response (UPR) of the host to its own advantage. The virus targets GRP78 for partial degradation to create a favorable environment for UPR induction and hijacks ATF4 into cytoplasmic replication complexes to promote viral RNA synthesis. The data also reveal potential targets (e.g., ATF4 and XBP1s) for antiviral drugs and have implications in vaccine development.

## Introduction

The unfolded protein response (UPR) in the endoplasmic reticulum (ER) represents an ancient but critical cellular mechanism in response to various deleterious stresses within the ER lumen. It maintains ER homeostasis by increasing protein-folding capacity, reducing global protein synthesis, and clearing misfolded proteins [1–3]. Protein loads and quality within the ER lumen are monitored by three transmembrane proteins: PKR-like ER kinase (PERK), activating transcriptional factor 6 (ATF6) and inositol requiring enzyme 1 (IRE1) [4,5]. These sensors are normally kept in an inactive state by GRP78 (also known as BiP or HSPA5), which is an ER-resident chaperone and the master regulator of UPR [6,7]. Under ER stress conditions, GRP78 detaches from the sensors to allow their activation as it proceeds to binds to exposed hydrophobic regions of nascent polypeptides [6,8].

The three sensors regulated by GRP78 control distinct pathways. Activated PERK phosphorylates eIF2α. Although this leads to global translational attenuation, it does allow the preferential translation of a subset of stress-related mRNAs, including the activating transcription factor 4 (ATF4) [9–11], which is a pro-survival protein that regulates the expression of stress-related genes involved in anti-oxidation, amino acid biosynthesis and transport, apoptosis, etc [12–15]. Sensor ATF6 can detect folding perturbations such as disruptions in disulfide bonding and protein under-glycosylation, and its activation leads to its translocation and cleavage within Golgi by host proteases to release a transcriptionally-active fragment that induces expression of genes involved in protein folding, such as GRP78, PDI and GRP94, etc [1,16–19]. Lastly, the IRE1-XBP1 branch is evolutionarily conserved from yeast to humans and responds to all types of ER stress [20,21]. Activated IRE1 has the endoribonuclease activity and splices many mRNA targets destined for translation at the ER, which causes IRE1-dependent mRNA decay [22,23]. Although most of the target mRNAs are degraded, the unconventional splicing of XBP1 mRNA produces a functional transcript that encodes the transcription factor XBP1s, which controls the expression of many target genes involved in lipid synthesis, ER-associated degradation (ERAD), and chaperones [24–26].

The cellular UPR has been increasingly recognized as an intrinsic line among innate defenses that can result in either degradation of microbial components, translational inhibition, or apoptosis [27–29]. Moreover, the UPR can regulate autophagy, inflammation, and type I interferon responses [30–32]. Consequently, investigations of how pathogens interface with this cellular response is required for a thorough understanding of how they replicate and cause disease [33]. The experiments described in this report address the interplay of UPR signaling with porcine reproductive and respiratory syndrome virus (PRRSV), a positive-strand RNA virus within the *Arteriviridae* family in the order *Nidovirales* [34,35].

PRRSV mainly causes reproductive failure of sows and respiratory diseases of young pigs, and it has remained a major threat to global pork production since the first outbreak in late 1980s [36]. Currently, there are no effective vaccines or anti-viral drugs available for this virus [37]. Persistent infections, dysregulation of host immunity, and rapid genetic mutation/recombination all contribute to the difficulty of disease control and the emergence of many highly virulent strains, including the deadly Chinese highly pathogenic PRRSV in 2006 [37–40]. Clearly, a better understanding of the interactions between this pathogen and its host is needed for developing effective prophylactic and therapeutic measures.

PRRSV has recently been shown to induce the activation of the PERK and IRE1 branches of the UPR in porcine alveolar macrophage cell line ZMAC [41], but the significance of these events for virus replication is unknown. Here, we show that the virus encodes a protein, GP2a, which targets GRP78 for degradation. Moreover, we show that the UPR does not inhibit but instead stimulates efficient replication of PRRSV. In particular, the virus appears to hijack ATF4 to viral replication and transcription complexes (RTCs) to facilitate viral RNA synthesis. Thus, our results reveal that PRRSV exploits UPR signaling for its own advantage.

## Results

### PRRSV targets the UPR master regulator GRP78 for degradation despite an increase at its mRNA level

**Fig 1:**
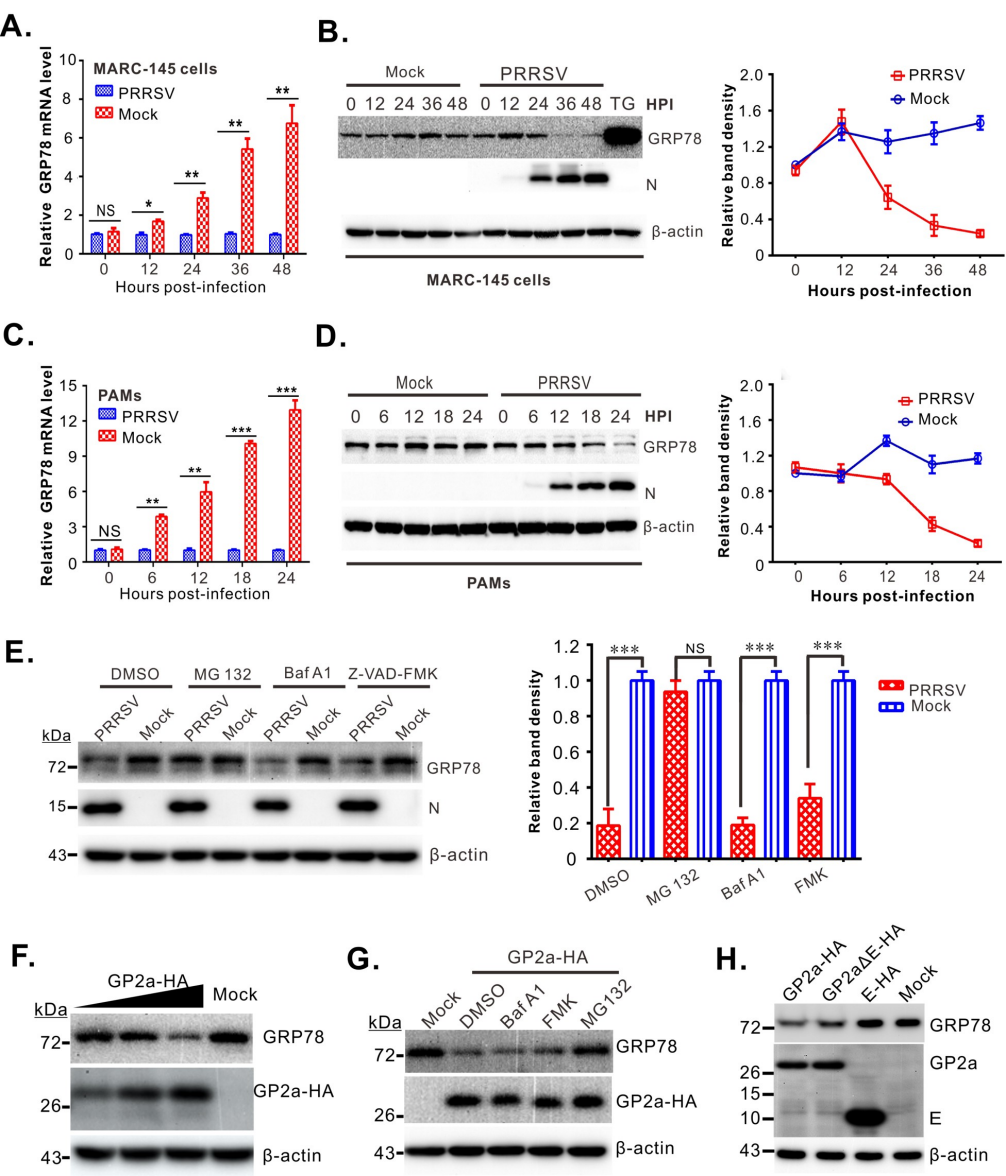
PRRSV targets GRP78 for proteasomal degradation via viral glycoprotein GP2a. (**A**) Relative abundance of GRP78 mRNA in PRRSV-infected MARC-145 cells at different time points post infection. The level of GRP78 mRNA was normalized against GAPDH and then compared to the uninfected group. (**B**) Western blot analysis of GRP78 protein levels in MARC-145 cells infected with PRRSV at an MOI of 0.1. TG treatment served as a positive control. β-actin served as a loading control, and the viral nucleocapsid protein, N, was used as an infection indicator. The graph shows the levels of GRP78 normalized against β-actin. (**C** and **D**) The same as (A) and (B), excepts that primary PAMs were used. (**E**) MARC-145 cells were infected with PRRSV strain JXwn06 at an MOI of 0.1, and at 24 hpi, the cells were treated with MG-132 (30 μM) or Bafilomycin A1 (0.5 μM) or Z-VAD-FMK (20 μM) for 2 h, before being collected for Western blot analyses. The graph shows the quantitative analysis of the GRP78 protein levels. (**F**) Decreasing levels of GRP78 in HEK-293FT cells in response to increasing levels of transiently-expressed PRRSV GP2a. (**G**) HEK-293FT cells grown on coverslips in six-well plates were transfected to express GP2a-HA. At 24 h post transfection, they were treated with MG-132 (30 μM), Bafilomycin A1 (0.5 μM), or Z-VAD-FMK (20 μM) for 6 h before being collected for western blot analyses. (**H**) HEK-293FT cells grown on coverslips in six-well plates were transfected to express GP2a-HA, GP2aΔE-HA, or E-HA. At 24 h post transfection, the cells were collected for Western blot analyses. Data information: Statistical analyses were performed by two-tailed Student’s *t*-test or one-way ANOVA, and asterisks (*) indicate the statistical significance: NS, no significance; *, *P* < 0.05; **, *P* < 0.01 ***; *P* < 0.001. Data are presented as means ± standard deviations (SD) of three independent experiments.

To investigate the mechanism of UPR induction by PRRSV, we initially focused on GRP78 because it is the master regulator that binds to and negatively controls sensors PERK, ATF6, and IRE1 [7,42]. When levels of misfolded proteins rise, GRP78 is released, resulting in activation of the UPR [7]. For many DNA and RNA viruses, their infections cause increased levels of GRP78, which is also a chaperone, so that the protein folding capacity of the cell can be enhanced [43–45]. In the case of PRRSV, we observed an elevation of GRP78 at the mRNA level in both PRRSV-infected MARC-145 cells (used for *in vitro* propagation of the virus; Fig 1A) and primary PAMs (porcine alveolar macrophages, the major *in vivo* cell target; Fig 1C). But in spite of these increases, we found that GRP78 protein levels actually declined as the infection proceeded (Fig 1B and 1D). In contrast, treatment with the potent ER-stress inducer thapsigargin (TG) resulted in a dramatic increase of GRP78 (Fig 1B), indicating that uninfected cells have the capacity to produce large amounts of this protein.

### The glycoprotein GP2a contributes to the GRP78 decay via proteasomal pathway

The mechanism of PRRSV-induced GRP78 decay was probed by treating mock or infected MARC-145 cells with either MG-132 (a proteasome inhibitor), Bafilomycin A1 (a lysosomal inhibitor), Z-VAD-FMK (an apoptosis inhibitor), or DMSO before collecting samples for analysis. Additions of DMSO, Bafilomycin A1, or Z-VAD-FMK did not have a significant effect, but treatment with MG-132 restored GRP78 to a level similar to that of uninfected cells (Fig 1E). Thus, the proteasomal degradation pathway contributes to the reduced levels of GRP78 seen in PRRSV-infected cells.

To identify the viral protein(s) needed for GRP78 decay, we used transfection assays in MARC-145 cells and HEK-293FT cells. Expression of nonstructural proteins (S1 Fig) and the major envelope membrane proteins (S2 Fig) did not decrease GRP78 expression but seemed to activate the UPR, as indicated by the apparently increased levels of this protein. In contrast, the minor envelope protein GP2a did not increase GRP78 expression (S2A Fig) but instead caused a decrease of this protein in about 50% of the cells (S2B Fig). This activity was confirmed by Western blot analyses in a dose-response experiment, which showed that increasing levels of GP2a caused decreasing levels of GRP78 (Fig 1F), and this effect could be reversed by treatment of the cells with MG-132 (Fig 1G). Because subgenomic mRNA2 contains the open reading frames for both GP2a and the E protein, we made constructs that eliminate the start codon for E (construct GP2aΔE-HA) or express only the E protein (E-HA). The results show that GP2a but not E protein decreased levels of GRP78 (Fig 1H).

### The downstream effectors ATF4 and XBP1s of UPR signaling are activated at an early stage of PRRSV infection

**Fig 2:**
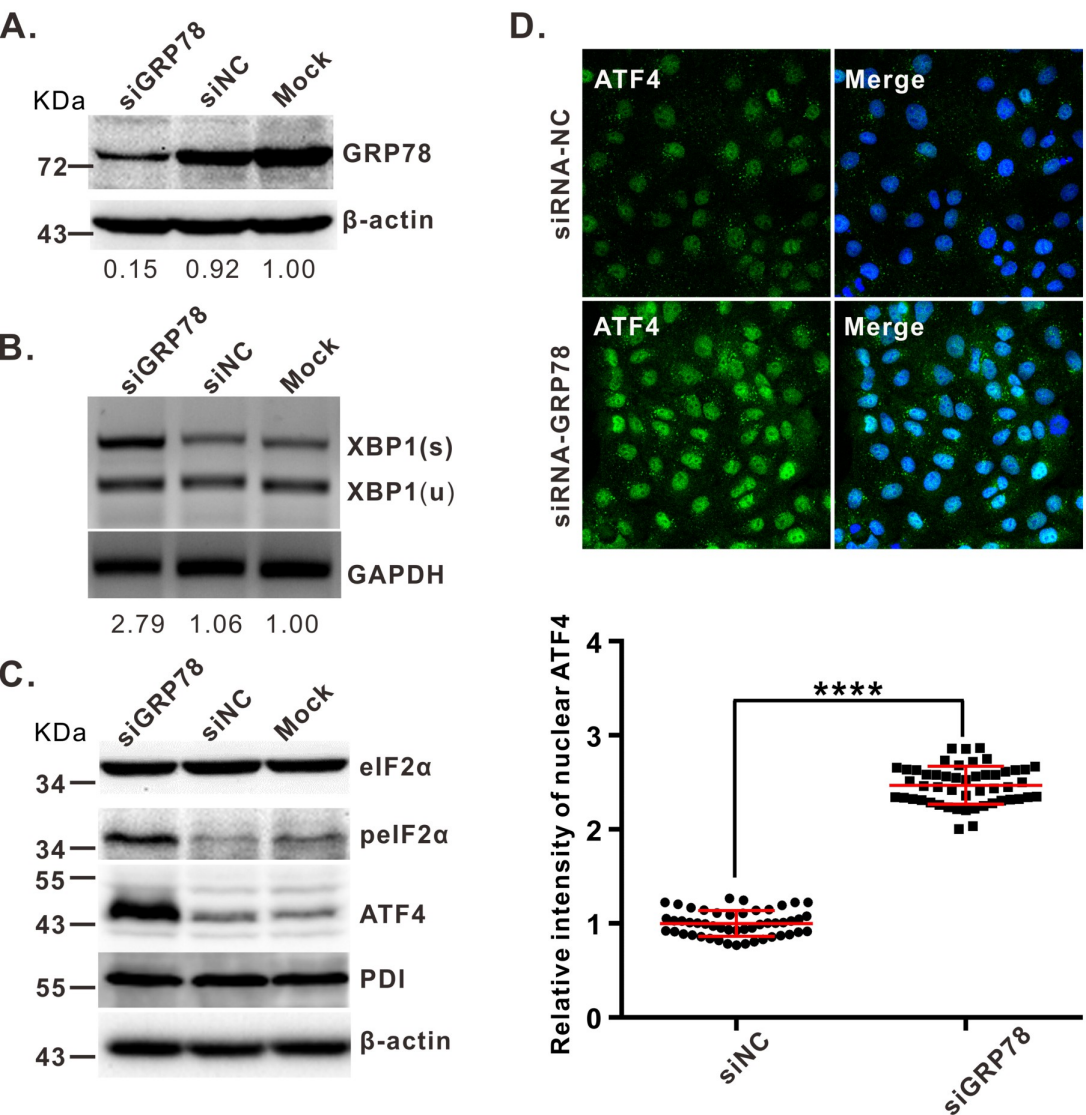
Knockdown of GRP78 activates ER stress. MARC-145 cells were transfected with siRNA targeting GRP78 (siGRP78) or scrambled siRNA (siNC). At 36 h post transfection, the cells were analyzed for UPR activation. (**A**) Analysis of knockdown efficiency of GRP78 by Western blot. (**B**) Detection of XBP1 mRNA splicing in siRNA-treated cells. The XBP1 gene was amplified by RT-PCR followed by enzyme digestion with *Pst I* and analyzed by separation on a 1.5% agarose gel. (**C**) Western blot analysis of the expression and phosphorylation status of indicated proteins. (**D**) Effect of GRP78 knockdown effect on the ATF4 activation by IFA staining. The relative fluorescence intensity of nuclear ATF4 were quantified by using image J (*N* = 50). Oil objective: 63 X; zoom in 1 X. Data information: Statistical analysis was performed by two-tailed Student’s *t*-test, and the data represent means ± standard deviations (SD). Asterisks (*) indicate the statistical significance: ***: *P* < 0.001; ****: *P*<0.0001; NS: no significance.

To verify that MARC-145 cells will exhibit the expected responses upon induction of the UPR, we transfected them with an siRNA that knockdowns expression of GRP78. In contrast to the mock-transfected control, RNAi silencing of GRP78 (Fig 2A) resulted in an increased amount of XBP1s (Fig 2B), elevated phosphorylation of eIF2α (Fig 2C), and increased expression of ATF4 (Fig 2C and 2D). As expected, the scrambled-sequence control (siNC) did not induce these effects.

**Fig 3:**
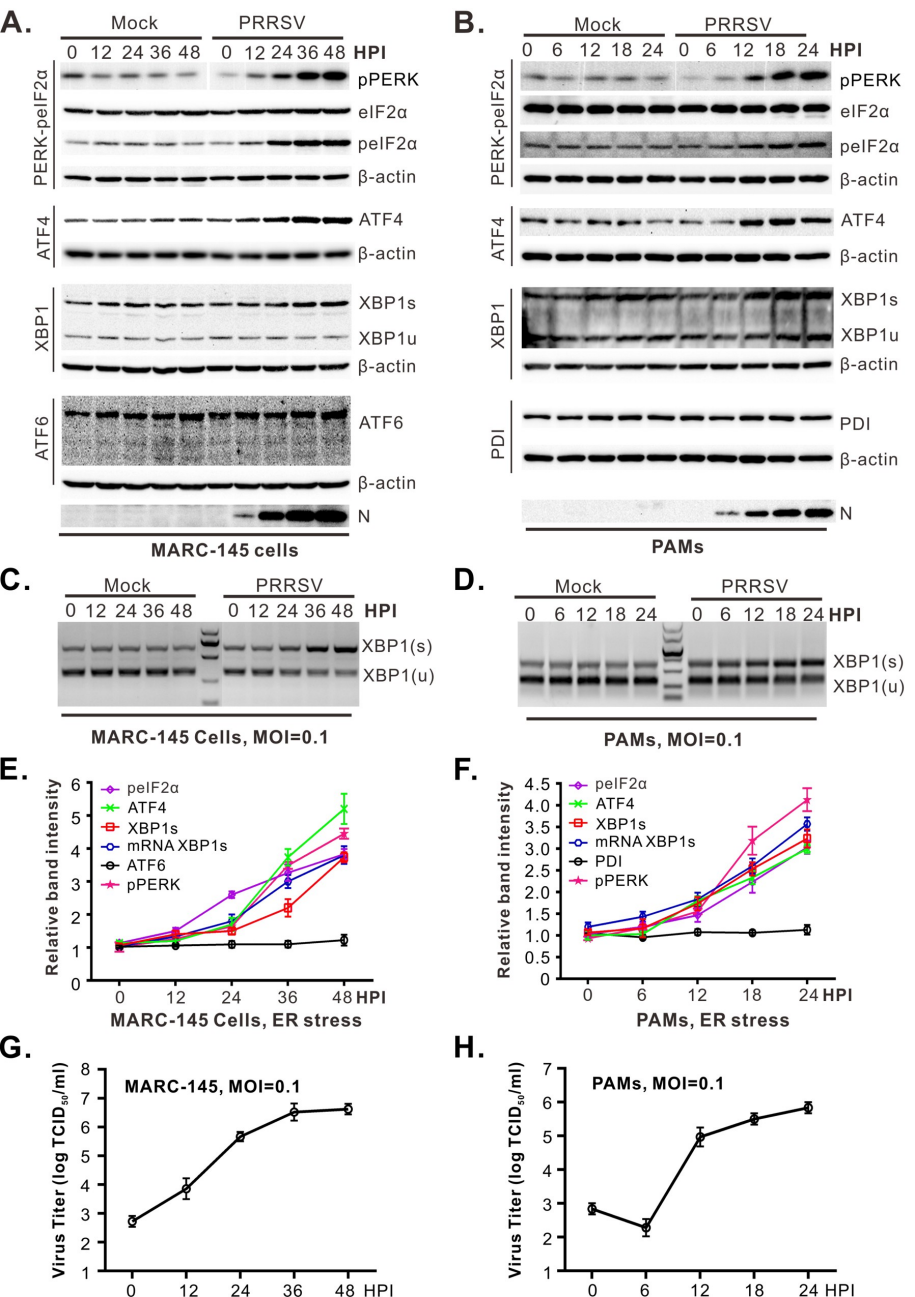
Downstream UPR effectors ATF4 and XBP1s are activated at an early stage of PRRSV infection. (**A** and **B**) MARC-145 cells or porcine alveolar macrophages (PAMs) were infected with PRRSV strain JXwn06 at an MOI of 0.1. At the indicated times post infection, the cells were collected and subject to western blot analysis with antibodies against the indicated proteins. Nucleocapsid protein N and β-actin served as the indicator for infection and the loading control, respectively. (**C** and **D**) Detection of XBP1 mRNA splicing. PRRSV-infected cells were harvested at the indicated times post infection. The XBP1 mRNA sequence was amplified by RT-PCR followed by digestion with *Pst* I and the products were analyzed by electrophoresis on a 1.5% agarose gel. (**E** and **F**) Quantification of the proteins or mRNAs during PRRSV infections. The abundance of pPERK, peIF2a, ATF4, XBP1s, ATF6, and PDI were expressed as fold changes compared to mock-infected control after being normalized against β-actin. (**G** and **H**) Kinetics of virus production in MARC-145 cells and PAMs at an MOI of 0.1. The results represent the averages of at least three independent experiments. Data information: Error bars indicate means ± standard deviations (SD).

While it seemed likely that the reduced levels of GRP78 in PRRSV infections (Fig 1B and 1D) would result in elevated expression of downstream components of the UPR, it was possible that these would be targeted for degradation, too. To investigate this, the expression levels of the various UPR components were examined. In MARC-145 cells, we found that both the PERK-eIF2α-ATF4 and IRE1-XBP1s branches of the UPR were activated (Fig 3A, 3C and 3E). In particular, there was a gradual increase in the phosphorylation of PERK and eIF2α, a concurrent increased in expression of ATF4, and an elevation of the spliced form (XBP1s) of XBP1 at both the protein and mRNA levels. In contrast, ATF6 was not activated, and the cleaved fragment of ATF6 was not detectable during infection. Similar results were observed in infected, primary PAMs (Fig 3B, 3D and 3F); however, the antibodies to ATF6 did not work on PAMs extracts, and PDI was used as a surrogate since its expression is subject to regulation by ATF6 [46].

The degradation of GRP78 and strong induction of downstream components raised the possibility that PRRSV might utilize some of the UPR machinery for its own benefit. To begin exploring this, we measured the temporal relationship between UPR induction and infectious virion production. Upregulation of ATF4 and XBP1s was detectable around 12 hpi in MARC-145 cells (Fig 3E) and 6 hpi in PAMs (Fig 3F). This coincided with detectable viral protein expression (here assayed for N; Fig 3A and 3B), but it occurred earlier than peak virus replication, which was at 36 hpi in MARC-145 cells (Fig 3G) and 24 hpi in PAMs (Fig 3H). The observed correlation of the timing of UPR induction and virus production in both cell types is consistent with the possibility of UPR machinery being needed for PRRSV replication.

### Both ATF4 and XBP1s are critical for PRRSV replication

**Fig 4:**
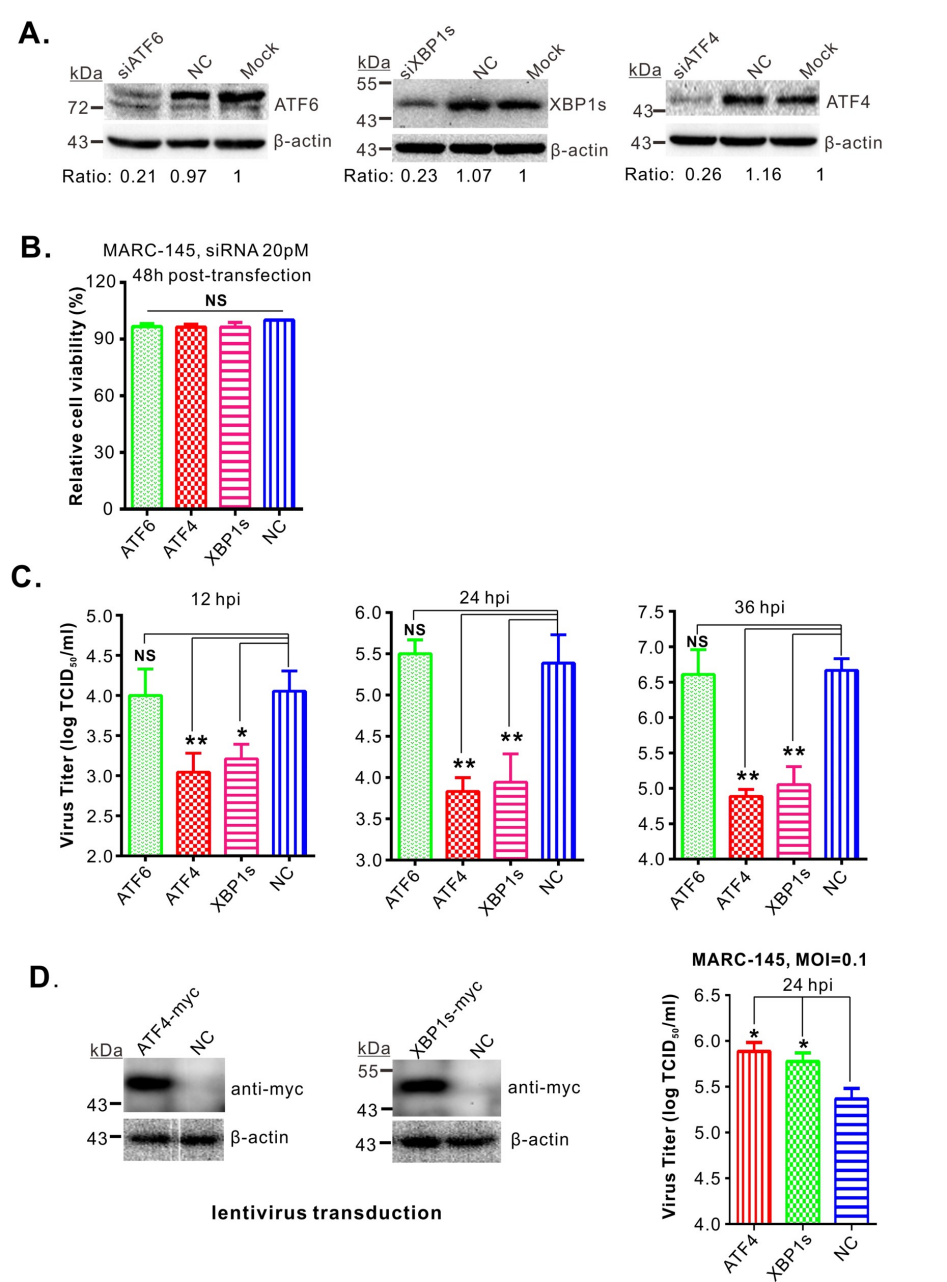
ATF4 and XBP1s are critical for PRRSV replication. (**A**) MARC-145 cells were transfected with indicated siRNA, and the knockdown efficiencies were assessed by Western blot at 36 h post transfection with the indicated antibodies. (**B**) Analysis of the cell viability at 36 h post siRNA transfection. (**C**) At 36 h post transfection with indicated siRNAs, MARC-145 cells were infected with PRRSV strain JXwn06 at an MOI of 0.1. At the indicated times, the total titer of infectious virus present in each culture was measured by the end-point dilution assay. (**D**) MARC-145 cells were transduced with lentivirus expressing ATF4-myc, XBP1s-myc, or the empty vector. At 36 h post transduction, the cells were infected with PRRSV at an MOI of 0.1. At 24 hpi, the total viral titer in each culture was measured by the end-point dilution assay (right panel), and the expression of the transduced proteins was analyzed by Western blot (left). Data information: Statistical analysis was performed by two-tailed Student’s *t*-test, and asterisks (*) indicate the statistical significance: NS, no significance; *, *P* < 0.05; **, *P* < 0.01; ***, *P* < 0.001. Data were presented as means ± standard deviations (SD) of three independent experiments.

The importance of both ATF4 and XBP1s for PRRSV replication was subsequently investigated with an RNAi knockdown approach. All siRNAs could efficiently silence the expression of the respective protein at 36 h post transfection (Fig 4A) without affecting cell viability (Fig 4B). Infection of these cells with PRRSV revealed a reduction in virus titers when either ATF4 or XBP1s expression was reduced, but depletion of ATF6 did not have this effect (Fig 4C). Also, overexpression of ATF4 or XBP1s in MARC-145 cells by means of lentivirus transduction significantly increased the virus yields (Fig 4D). Thus, both ATF4 and XBP1s, two downstream effectors of the UPR, are critical for PRRSV replication.

### ATF4 is hijacked to PRRSV replication complexes during infection

**Fig 5:**
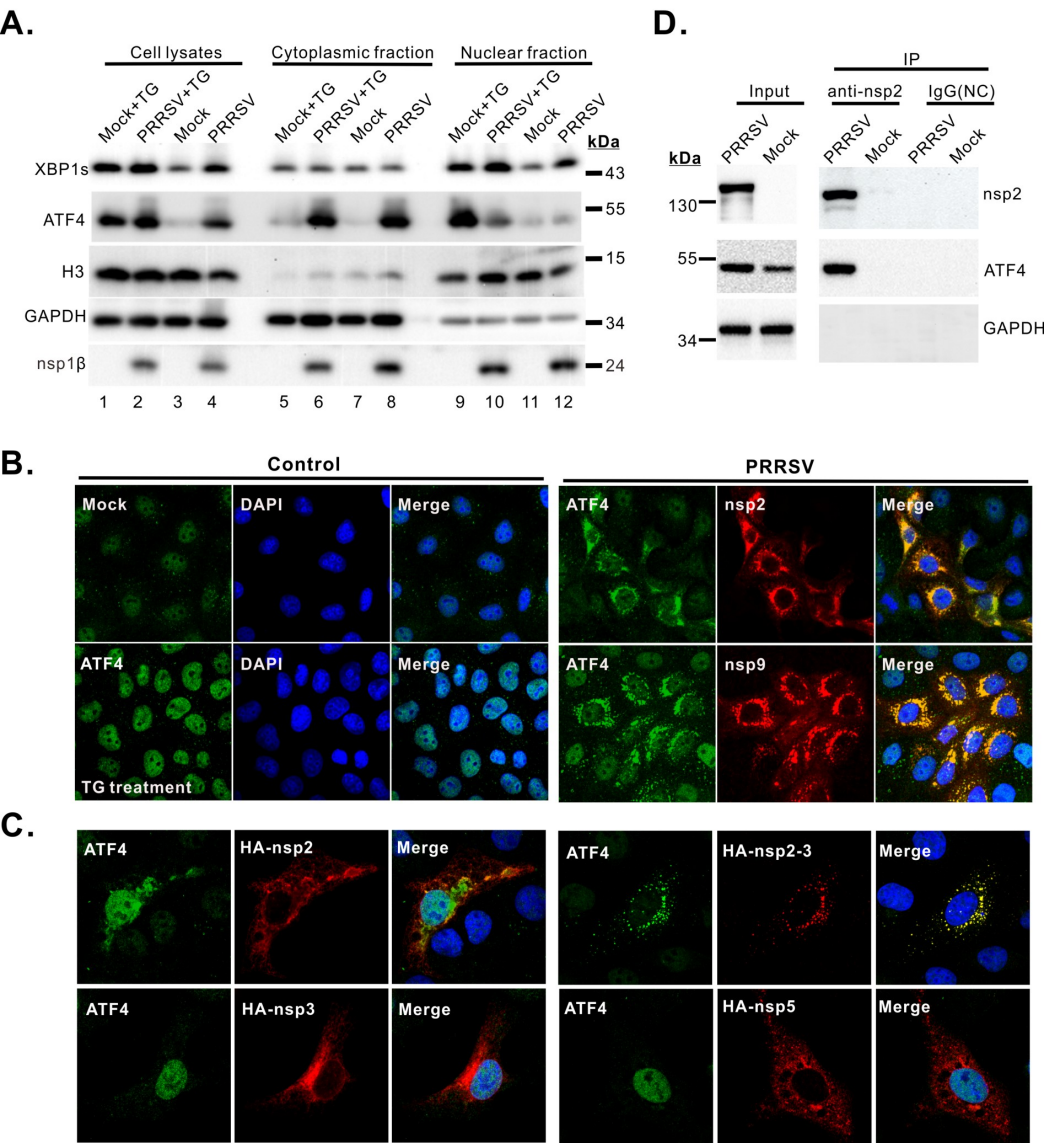
PRRSV nonstructural proteins nsp2/3 hijack ATF4 to viral replication complexes. (**A**) MARC-145 cells were mock-infected or infected with PRRSV strain JXwn06 at an MOI of 0.1. At 36 hpi, the cells were treated or untreated with TG (200 nM) for 0.5 h. The cell cytoplasmic and nuclear fractions were prepared and assayed for the presence of XBP1s and ATF4. H3 and GAPDH were used as fractionation quality control and nsp1β was used as an infection indicator. (**B**) Confocal microscopy to locate ATF4 in infected cells. Control cells: ATF4 (green) localization in MARC-145 cells untreated or treated with TG (200 nM, 0.5 h) as negative and positive control. PRRSV infected cells: The position of ATF4 (green) relative to nsp2 (red) and nsp9 (red) in virus-infected cells at 24 hpi (MOI = 0.1). Oil objective: 100 X; zoom in 1 X. (**C**) TG-treated MARC-145 cells were examined by confocal microscopy to look for retention of ATF4 (green) in the cytoplasm. Oil objective: 100 X; zoom in 2 X. (**D**) Co-IP analysis of the interaction between endogenous ATF4 and nsp2 in PRRSV-infected MARC-145 cells at 36 hpi (MOI = 0.1). A mouse isotype antibody used as a control. Data information: The data were acquired by Nikon A1 confocal microscope and represent at least three independent experiments.

To explore the roles of ATF4 and XBP1s in PRRSV replication, we began by using a cytoplasmic-nuclear fractionation assay to measure their cellular distribution. MARC-145 cells were either infected with PRRSV or mock infected, and duplicates of these cultures were then treated with either thapsigargin (TG) or DMSO at 24 hpi for 0.5 h. The cytoplasmic and nuclear fractions were isolated and probed for the presence of ATF4 and XBP1s (Fig 5A). The quality of the fractionation was checked by assaying for cytoplasmic GAPDH and nuclear histone subunit H3. As expected, TG-treatment and PRRSV infection upregulated the expression of both ATF4 and XBP1s (Fig 5A, compare lanes 1 and 4 with lane 3). In the case of XBP1s, the protein was enriched in the nuclear fraction whether the cells were treated with TG (compare lanes 5 and 9) or infected (compare lanes 8 and 12). To our surprise, ATF4 behaved differently. In TG-treated cells, this protein was almost exclusively found in the nuclear fraction (compare lanes 5 and 9), but in infected cells, it was found almost exclusively in the cytoplasmic fraction (compare lanes 8 and 12). In addition, PRRSV infection blocked the TG-induced ATF4 nuclear translocation (Fig 5A, compare lanes 6 and 10). Thus, PRRSV appears to have a mechanism for retaining ATF4 in the cytoplasm.

To verify these observations *in situ*, an immunofluorescence assay (IFA) was used to detect the subcellular location of ATF4. (Unfortunately, the antibodies against XBP1s did not work in this assay). We found that ATF4 was not within nuclei but instead was present at perinuclear regions in PRRSV-infected cells (Fig 5B, right panels), and the TG-induced ATF4 nuclear translocation was blocked by PRRSV infection (S3A Fig). Moreover, ATF4 was found to co-localize very well with PRRSV nsp2 and nsp9 (Fig 5B, right panels), which are key components of viral replication and transcription complexes (RTCs) [47], suggesting that ATF4 is hijacked to viral RTCs during infection. Similar results were obtained in PRRSV-infected primary PAMs (S4 Fig). Consistent with cytoplasmic retention, the canonical ATF4-target genes GADD34 and ASNS [48] were not upregulated during infection as compared with the mock or TG-treated cells (S3B and S3C Fig). Also, we found that this phenomenon is independent of PRRSV strain used (S5 Fig) and unique to PRRSV (S6 Fig), since it was not observed with several other RNA viruses, including equine arteritis virus (EAV), encephalomyocarditis virus (EMCV), classical swine fever virus (CSFV), and porcine epidemic diarrhea virus (PEDV).

### PRRSV nsp2/3 is sufficient to retain ATF4 in the cytoplasm

To gain insight to how ATF4 is diverted to viral RTCs, individual nonstructural proteins (nsps) were screened for their ability to cause cytoplasmic retention of ATF4 in MARC-145 cells that were treated with TG for 0.5 h at 24 h post-transfection (S7A Fig). We found that PRRSV nsp2 was able to partially retain ATF4 in the cytoplasm (Fig 5C and S7B Fig), but co-expression with nsp3, which is known to form heterodimer with nsp2, dramatically increased the retention efficiency, even though nsp3 by itself—like nsp5—did not have any activity (Fig 5C). The interaction between nsp2 and endogenous ATF4 was also confirmed with a co-IP assay using infected cell lysates (Fig 5D). These results indicate that nsp2/3 contributes to the recruitment of ATF4, which is also consistent with the proposed role of nsp2/3 as membrane scaffolding proteins for viral RTCs assembly [49,50].

### Recruitment of ATF4 to viral RTCs is an early event in PRRSV infection

**Fig 6:**
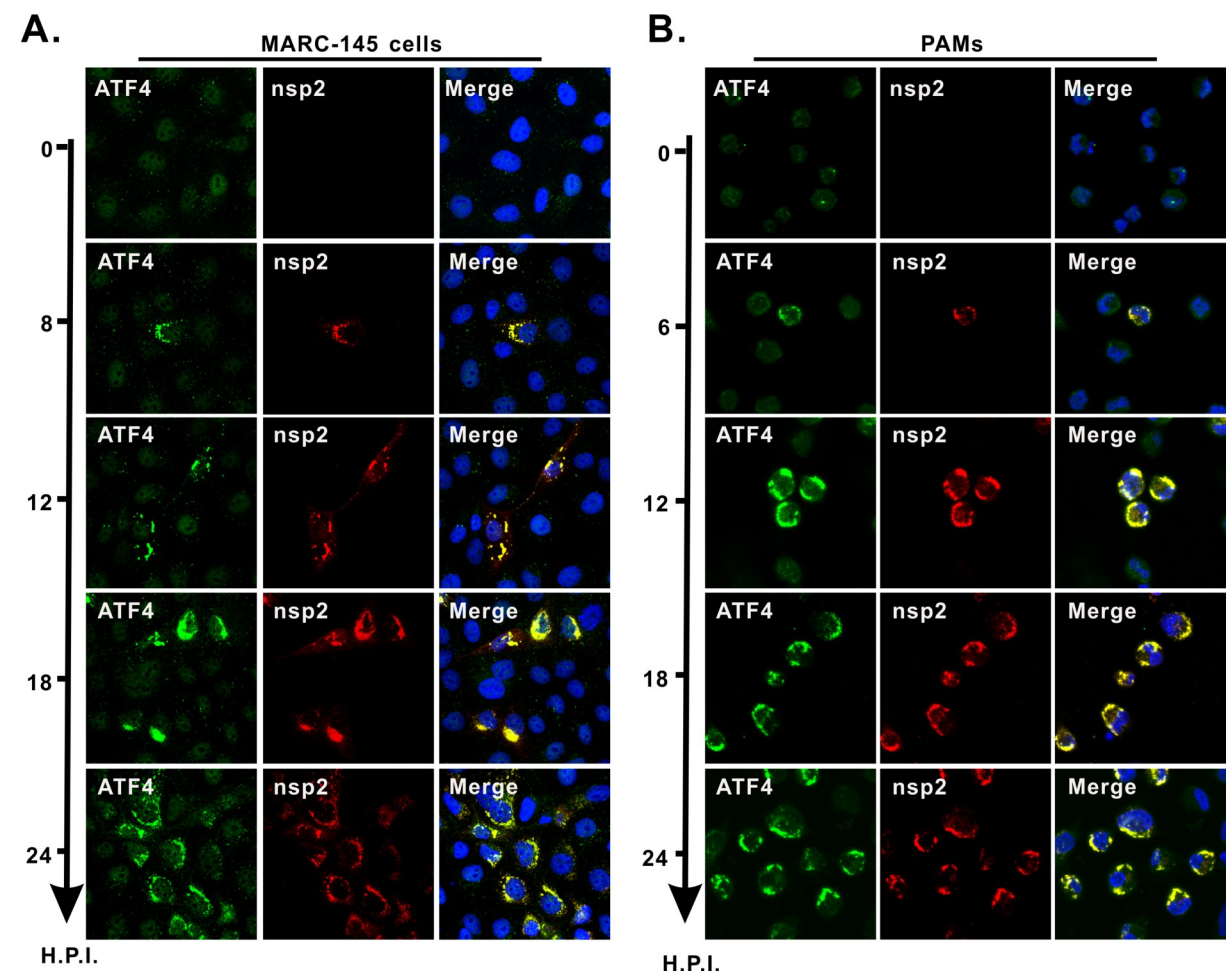
Recruitment of ATF4 to viral RTCs is an early event. (**A** and **B**) MARC-145 cells or PAMs grown on coverslips in six-well plates were infected with PRRSV strain JXwn06 at an MOI of 0.1. At the indicated times, the cells were fixed, permeablized, and stained with the antibodies against ATF4 and nsp2. Data information: The images were acquired by Nikon A1 confocal microscope and are representative of at least three independent experiments. Oil objective: 100 X; zoom in 1 X (A) and 2 X (B).

To learn when ATF4 is redirected to viral RTCs, we used immunofluorescence staining at different times after infection. The results showed that ATF4 became detectable around 8 hpi in MARC-145 cells, concurrent with the expression of the nsp2 replicase protein, and both co-localized well at the perinuclear region (Fig 6A). Similar finding was observed in primary PAMs (Fig 6B). The detection of ATF4 was earlier than observed with Western blots (Fig 3), and this is likely because a low MOI was used (0.1). Because most cells are not infected, the IFA would be expected to have greater sensitivity because it enables the detection of protein expression at the single cell level. In any case, it is clear that recruitment of ATF4 to viral RTCs is an early event in PRRSV infection.

### ATF4 interacts with PRRSV RNA and promotes viral RNA accumulation

**Fig 7:**
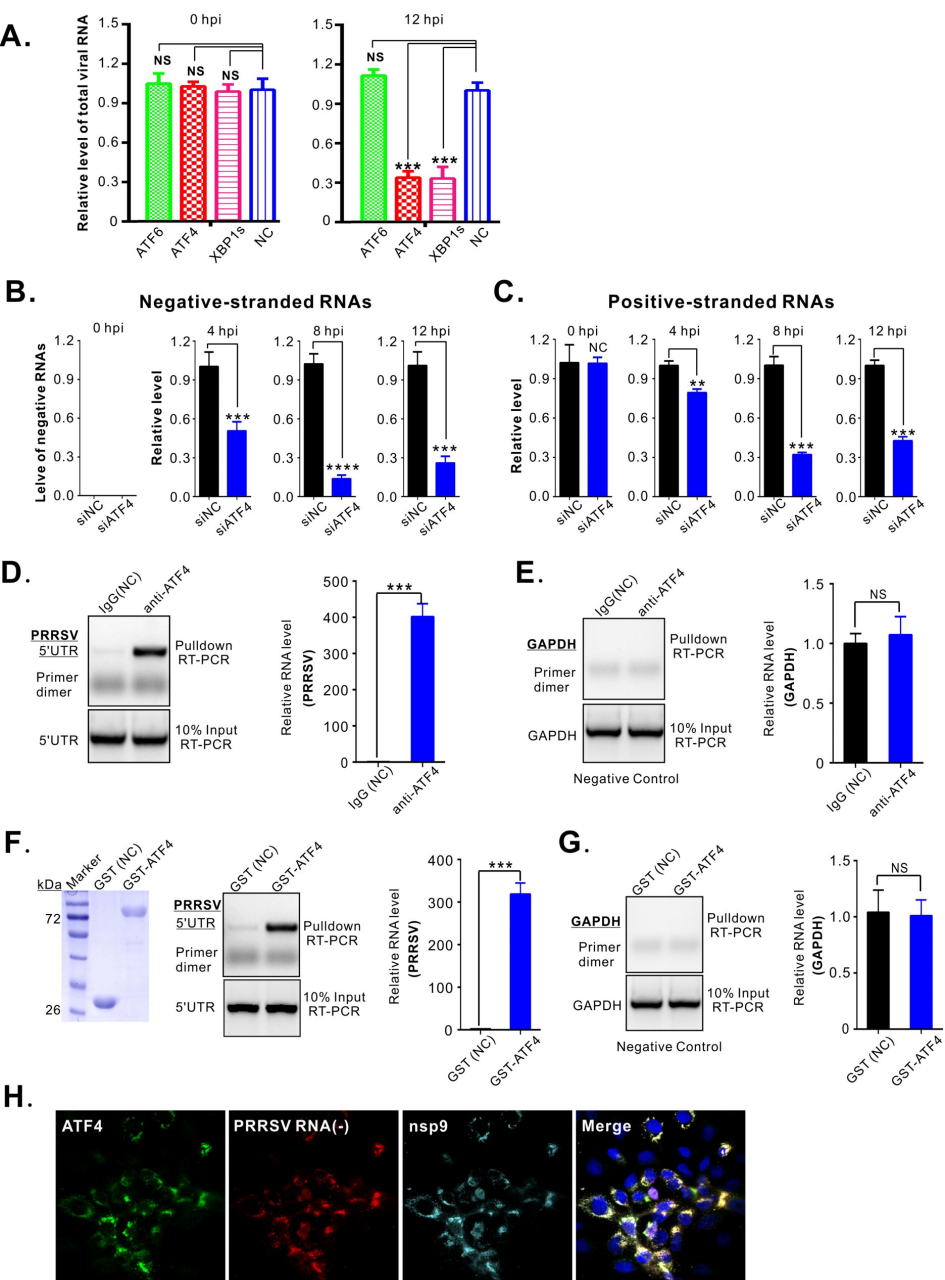
ATF4 interacts with PRRSV RNA and promotes its accumulation. (**A**) MARC-145 cells were transfected with an siRNA targeting ATF4 (siATF4) or scrambled siRNA (siNC). At 36 h post transfection, the cells were infected with PRRSV strain JXwn06 at an MOI of 1. The abundance of viral RNA was assessed by RT-PCR of ORF7, normalized against the house-keeping gene GAPDH, and then compared to siNC control at 0 to 12 hpi. (**B** and **C**) The same as (A) except the abundance of positive- and negative stranded RNAs were analyzed by RT-qPCR at 0, 4, 8 and 12 hpi. (**D**) Control, rabbit IgG or antibodies specific for ATF4 were used for immunoprecipitations from lysates of infected MARC-145 cells (MOI = 0.1, 36 hpi). The presence of viral RNA was assayed by RT-qPCR targeting 5’-UTR sequence or (**E**) GAPDH as a negative control, and the fold enrichment was calculated against the normal rabbit IgG group. (**F**) GST-ATF4 or GST were purified from *E*. *coli* (left panel) and added to RNA extracted from PRRSV-infected MARC-145 cells in an *in vitro* binding assay. RT-qPCR with primers specific for a 5’UTR sequence was used to detect viral RNA (middle panel) and the fold of enrichment of viral RNA was expressed against the GST control. (**G**) In parallel, the pull down of cellular GAPDH mRNA was used as a negative control. (**H**) To look for ATF4 within viral replication compartments in infected MARC-145 cells (MOI = 0.1, 24 hpi), the negative-strand RNA was detected by the RNAscope *in situ* hybridization method, and ATF4 and nsp9 were detected by immunostaining. Data information: Statistical analysis was performed by two-tailed Student’s *t*-test, and error bars indicate standard deviations (SD) of means. Asterisks (*) indicate the statistical significance: ***, *P* < 0.001; ****, *P*<0.0001; NS, no significance (*n* = 3 experiments in each condition). Confocal images were acquired with Nikon A1 confocal microscope. Oil objective: 63 X; zoom in 1 X.

Having found that ATF4 is critical for high virus titers and co-localizes with viral RTCs in PRRSV infections, we hypothesized that this component of the UPR facilitates viral RNA synthesis. Consistent with this, RNAi knockdown of ATF4 greatly reduced the level of total viral RNAs, as measured by RT-qPCR analysis (Fig 7A), and this correlated well with the impaired virus production (Fig 4C). To investigate which step of viral RNA synthesis was affected, we measured the relative abundance of negative- and positive-stranded RNAs in MARC-145 cells that had been transfected with either siRNA-ATF4 or the scrambled siNC 36 h prior to infection. RNA levels were analyzed during a single viral replication cycle (i.e., within 12 hours) by RT-qPCR amplifying ORF7 RNA and calculating the amount present relative to the RNA from GAPDH, a house-keeping gene. The relative abundance of each viral RNA was then calculated as the ratio of the siRNA-ATF4-treated group compared to the siNC-treated control. As expected, no negative-stranded viral RNAs could be detected in either the control or siATF4-treated cells at 0 hpi (Fig 7B). At 4–12 hpi, this RNA became detectable, but its accumulation was greatly reduced when ATF4 was depleted. Positive-stranded RNA could be detected at 0 hpi due to the input of virions used to start the infection, but a gradual, obvious decrease was observed over time (Fig 7C). We also measured the abundance of all subgenomic mRNAs with primers that specifically amplify the leader-body junctions. The results show that reduced ATF4 expression had an effect on all RNA species regardless of the strands (S8A and S8B Fig), suggesting that this protein is a host factor required for viral RNA synthesis.

In support of our hypothesis, antibodies to ATF4, but not the isotype control (IgG), could immunoprecipitate viral RNA from infected MARC-145 cells, as detected by RT-qPCR of the PRRSV 5’UTR region (Fig 7D). Also, a purified GST-ATF4 chimera, but not GST alone, could pull down viral RNA from purified infected-cell RNA (Fig 7F), which suggests that an ATF4-RNA interaction can occur in the absence of other viral factors. As negative controls, neither the ATF4 antibodies nor the GST-ATF4 chimera pulled down GAPDH RNA (Fig 7E and 7G). Lastly, confocal microscopy of infected cells showed that ATF4 exhibited remarkable co-localization with negative-stranded viral RNA (detected by RNAscope *in situ* hybridization), as well as the viral polymerase nsp9 in infected cells (Fig 7H). Together, the above results provide strong evidence for a critical role of ATF4 in PRRSV RNA synthesis via an interaction with viral RNA.

## Discussion

**Fig 8:**
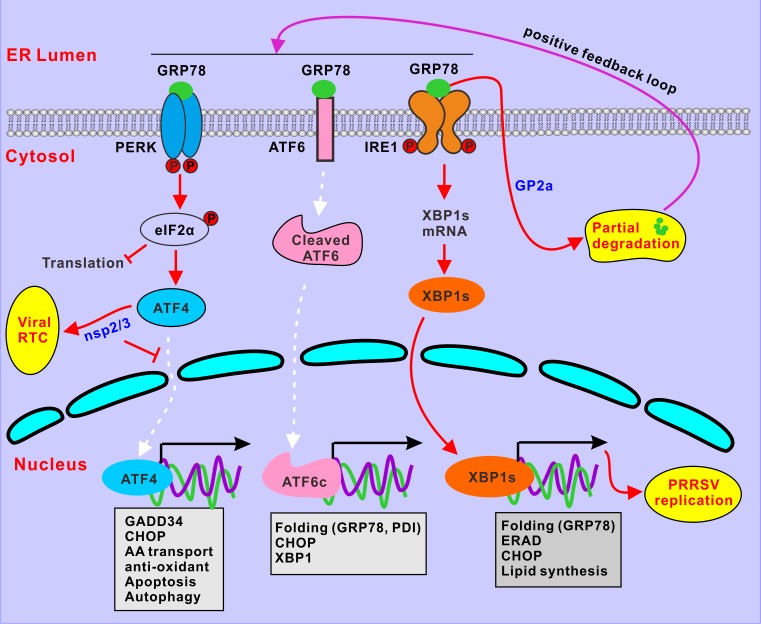
A proposed model for PRRSV manipulation of the cellular UPR. PRRSV turns on UPR at early stage of infection via both PERK-eIF2α-ATF4 and IRE1-XBP1s branches. The activated effector XBP1s enters nucleus, but ATF4 is diverted to viral replication complexes by nonstructural proteins nsp2/3 to promote viral RNA synthesis. To maintain a favorable environment, PRRSV targets GRP78 for partial proteasomal degradation by viral glycoprotein GP2a, which creates a positive feedback loop to sensitize and potentiate the UPR signaling.

The endoplasmic reticulum has complex roles in autophagy, apoptosis, and innate immunity, and thus, is an important organelle in viral pathogenesis [34]. This is also the location where the UPR originates, and previous studies have revealed an association of this response with PRRSV-induced apoptosis and dysregulation of TNF-α production in cell culture [41,51]. In this study, two unexpected findings were made that together reveal a novel way in which PRRSV reprograms the cellular UPR to its own advantage. In particular, the virus targets GRP78 for degradation to induce or potentiate the UPR, and it then hijacks ATF4 to viral replication complexes to promote viral RNA replication. A proposed model for PRRSV manipulation of the cellular UPR is shown in Fig 8.

### Degradation of GRP78, the UPR regulator and chaperone

The first unexpected finding from this study was the downregulation of GRP78 in a manner that is proteasome dependent and requires a viral membrane protein GP2a. It is likely that the ERAD pathway is utilized to retrotranslocate GPR78 into the cytosol, where proteasomes reside [52], but the details of how GP2a might cause this remain to be elucidated. In any case, downregulation of GRP78 was surprising because many viruses, especially enveloped viruses and ER-tropic viruses (e.g., the flaviviruses and coronaviruses), quickly produce large amounts of membrane proteins and thereby place a large burden on the folding machinery in the ER [53–55]. In response to this stress, most viruses tend to upregulate chaperon GRP78 and other factors to meet the increased demand for folding and post-translational modifications [56]. This is not the case for PRRSV. Because GRP78 is the master regulator, its reduced accumulation likely prevents any modulation of the UPR, keeping the ER stress pathways induced for the benefit of the virus. In addition to providing downstream factors such as ATF4 that this virus utilizes for replication, the reduced levels of GRP78 will likely result in increased levels of misfolded cellular proteins, potentially aiding in immune evasion [42,57,58]. What remains unclear is how PRRSV promotes the folding of its own proteins in this situation.

### Repurposing ATF4 for the replication of PRRSV

The second unexpected, and perhaps most exciting, finding from this study is the dependence of PRRSV on two downstream UPR effectors, ATF4 and XBP1s, which were induced in the reduction of GPR78. With regard to the PERK-eIF2α-ATF4 signaling branch of the UPR, previous studies of how viruses utilize ATF4 have only revealed effects that depend upon its activity as a DNA transcription factor. For example, in the cases of West Nile virus (WNV) and Infectious Bronchitis virus (IBV) [59,60], ATF4 upregulates transcription factor CHOP to induce apoptosis and virus release. In these cases, activation takes place during the late stages of infection, after high titers of viruses have been made. Other viruses (e.g., HSV-1 and dengue virus) use ATF4 to upregulate GADD34 to antagonize eIF2α phosphorylation [61,62]. In the case of retroviruses (e.g., HIV), ATF4 has been found to be diverted and bound to viral promoters to mediate transcriptional activation, and thus virus replication [63].

In our studies, ATF4 was found to be sequestered in the cytoplasm during PRRSV infections, and consistent with this observation, the canonical ATF4 target genes such as GADD34 and ASNS [51] were not upregulated and eIF2α remained phosphorylated. This property of PRRSV appears to be similar to that of mouse hepatitis virus (MHV) [64], where high levels of phosphorylated eIF2α and activation of ATF4 were found without subsequent expression of GADD34; however, in that work, the authors did not examine possible roles for, or the intracellular location of, ATF4.

Mechanistically, we found that ATF4 is recruited by nsp2/3 to viral RTCs to promote viral replication. When this protein was depleted with siRNAs, the accumulation of viral RNA was severely attenuated for both the positive and negative strands. Indeed, the effect seems to be equal on the accumulation of all viral RNA species at the transcriptional level, suggesting that ATF4 serves as a general facilitator for viral RNA biogenesis. This is unlike host RNA helicase DDX1, which is relocated to the cytoplasm in coronavirus infections [65] and is needed for the synthesis of much longer viral RNA species [66]. Further evidence supporting the association of ATF4 with the replication machinery of PRRSV includes its interaction with viral RNA in pulldown assays and co-localization with negative-strand RNAs during infection. Whatever its exact role, depletion of this component of the UPR led to a dramatic attenuation of PRRSV titers, whereas overexpression increased the viral yield. Future studies are needed to dissect how exactly ATF4 promotes RNA biogenesis and to identify the ATF4-interacting domain or motif within PRRSV so that a mutant virus can be tested for vaccine development.

Interestingly, ATF4 has also been implicated in cancer progression [67]. In tumors, ATF4 expression is detected in hypoxic- and nutrient-deprived regions where it promotes metabolic homeostasis and cancer cell survival by transcriptionally regulating amino acid uptake and biosynthesis, autophagy, redox balance and angiogenesis [15,67–69]. As ATF4 is normally expressed at low levels and is not essential for normal cells, this molecule serves as a potential target for drug development in both human and veterinary medicine. Currently, there are no approved drugs for this target, and a search for one is warranted.

We also found that PRRSV is dependent on XBP1s. The IRE1 branch of the UPR is linked to many functions, such as apoptosis through the IRE1-mediated RIDD and JNK pathways, inflammation through IRE1-ASK pathway, autophagy, ERAD, and lipid synthesis through activation of XBP1s [23,24,26,70,71]. Our study showed that IRE1 is activated in infected cells, consistent with two previous reports [41,51]. While it is clear that the downstream effector XBP1s is important for virus replication the mechanism was not further explored here. There are several ways that XBP1s might be important. One is for fulfilling the increased demand for lipid synthesis, as the replication of some viruses, including PRRSV, often involves extensive modulation, rearrangement and capture of intracellular membranes into virions [24,25,72]. Second, XBP1s may be important for regulation of autophagy [71]. Indeed, XBP1s has been shown to be an important regulator of genes involved in autophagy biogenesis, such as LC3, ULK1, ATG4, Beclin1, and Bcl2 [31,71,73]. Third, XBP1s may facilitate the degradation of misfolded proteins via the ERAD pathway and thereby increase the folding capability via protein chaperones [5,74]. PRRSV appears to be different from closely-related coronaviruses in its requirement for XBP1s signaling. For example, MHV induces splicing of XBP1 but suppresses the activation of its downstream target genes [64]. Also, transmissible gastroenteritis virus (TGEV) triggers the activation of IRE1-XBP1s signaling, but this pathway is not required for viral replication [75]. In any case, since XBP1s is a stress-inducing gene product and normally expressed at low level, it represents a potential antiviral target for PRRSV.

In summary, the findings reported here provide further insight into the mechanisms by which PRRSV interacts with cellular pathways and reveal a novel paradigm for how pathogens can repurpose components UPR for their own use. Our results also identify potentially druggable targets (ATF4 and XBP1s) and provide new information for making attenuated vaccines to control PRRSV.

## Materials and methods

### Ethics statement

The sampling of primary porcine pulmonary alveolar macrophages (PAMs) derived from one-month-old SPF pigs was performed according to the Chinese Regulations of Laboratory Animals—*The Guidelines for the Care of Laboratory Animals* (Ministry of Science and Technology of People's Republic of China) and Laboratory Animal-Requirements of Environment and Housing Facilities (GB 14925±2010, National Laboratory Animal Standardization Technical Committee). The license number associated with this research protocol was CAU 20120611, which was approved by the Laboratory Animal Ethical Committee of China Agricultural University.

### Viruses and cells

The Chinese highly pathogenic PRRSV strain JXwn06 (GenBank accession no: EF641008), low pathogenic PRRSV strain HB1/3.9 (EU360130), the NADC30-like PRRSV CHsx1401 (KP861625) [76,77], Porcine epidemic diarrhea virus (PEDV) BJ2011C strain [78], and Encephalomyocarditis virus (EMCV) strain (BJC3) [79] have been documented previously. Classical swine fever virus (CSFV) C strain was a gift from by Dr. Qin Wang (China Institute of Veterinary Drug Control). The equine arteritis virus (EAV) infectious clone was kindly provided by Dr. Eric J. Snijder (Leiden University Medical Center, Netherland) [80]. ST cells (Swine testicle) (ATCC CRL-1746) provided by Dr. Qin Wang (China Institute of Veterinary Drug Control) were derived from swine testicles. MARC-145 cells (African green monkey kidney epithelial cells) (ATCC CRL-12231), Vero cells (African green monkey kidney epithelial cells) (ATCC CRL-1586), BHK-21 cells (Baby hamster kidney) (ATCC CRL-12071), and HEK-293FT cells (Human embryonic kidney cells) (Thermo, #R70007) were all cultured in Dulbecco’s modified Eagle’s medium (DMEM) (Gibco, #12491015) with 10% fetal bovine serum (FBS) (Gibco, #16140071) and penicillin (50 U/mL) & streptomycin (50 μg/mL) at 37°C in a humidified atmosphere of 5% CO_2_. Primary porcine pulmonary alveolar macrophages (PAMs) derived from one-month old SPF pigs were maintained in RPMI-1640 (Gibco, #61870044) medium, containing 10% FBS and penicillin (50 U/mL) & streptomycin (50 μg/mL).

### Antibodies and chemicals

Antibodies used in this study were obtained from a variety of sources. Mouse anti-HA monoclonal antibody (MAb) (#H3663), rabbit anti-Myc polyclonal antibody (PAb) (#C3956) and mouse anti-β-actin MAb (#A5441) were all purchased from Sigma–Aldrich (MO, USA). Rabbit anti-GRP78 MAb (#cs-3177), rabbit anti-PDI PAb (#2446), rabbit anti-PERK MAb (#3192), rabbit anti-eIF2α PAb (#cs-5324), and rabbit anti-phospho-eIF2α (Ser51) MAb (#cs-5199) were obtained from Cell Signaling Technology (Beverly, MA). Rabbit anti-ATF4 PAb (#NBP2-15499) and rabbit anti-XBP1 PAb (#NBP2-20917) were obtained from Novus Biologicals (Littleton, USA). Rabbit anti-ATF6 PAb (#24169-1-AP), anti-GADD34 PAb (#10449-1-AP), anti-GAPDH PAb (#10494-1-AP) and Histone-H3 PAb (#17168-1-AP) were purchased from Proteintech (Chicago, IL). Normal mouse IgG used as negative control was purchased from Beyotime (#A7028). Mouse anti-N (PRRSV), anti-nsp1β (PRRSV), anti-nsp2 (PRRSV), anti-nsp9 (PRRSV), mouse anti-N (PEDV) and anti-VP2 (EMCV) MAb were produced by our lab at China Agricultural University and used as previously described [81,82]. Mouse anti-dsRNA MAb J2 (#10010500) was purchased from SCICONS. Mouse anti-E2 (CSFV) MAb was gifted by Dr. Qin Wang (China Institute of Veterinary Drug Control). Alexa Fluor 568-conjugatd goat anti-Mouse F(ab')2 fragment (#11019), Alexa Fluor 488-conjugated goat anti-mouse F(ab')2 fragment (#11017), Alexa Fluor 488-conjugated goat anti-rabbit F(ab')2 fragment (#11070), Alexa Fluor 647-conjugated goat anti-rabbit F(ab’)2 fragment (#21246), and Alexa Fluor 568-conjugate goat anti-rabbit F(ab') fragment (#11011) were obtained from Thermo Fisher. Protease inhibitors cocktail (#P8340), MG-132 (#S2619), Bafilomycin A1 (#B1793), Thapsigargin (#T9033) and DL-Dithiothreitol solution (#43816) were obtained from Sigma–Aldrich.

### Plasmid construction and transfection

The plasmids expressing nsp1α, nsp1β, nsp2, nsp3, nsp4, nsp5, nsp7, nsp8, nsp9, nsp10, nsp11, nsp12, GP2a, GP3, GP4, GP5, M and N were constructed by cloning the corresponding coding region from PRRSV strain JXwn06 genome into the vector pCMV-HA (Clontech, #631604). GP2aΔE-HA was created by introducing the corresponding mutations into the plasmid pGP2a-HA with the QuikChange Site-Directed Mutagenesis Kit (Agilent Technologies, #200523). The ATF4 gene from MARC-145 cells was cloned into the vector pGEX-6P-1 (GE healthcare #28-9546-48) for expression of the fusion protein GST-ATF4 in *E*. *coli* BL21 cells (Takara, #9126). Transfection of plasmid was performed with Lipofectamine^™^ LTX Reagent (Thermo Fisher, #A12621) according to the manufacturer’s instructions.

### Preparation of nuclear and cytoplasmic fractions

MARC-145 cells were infected with PRRSV strain JXwn06 at an MOI of 0.1. At 36 hpi, the cells were washed twice with cold phosphate-buffered saline (PBS) (140 mM NaCl, 2.7 mM KCl, 10 mM Na_2_HPO_4_, 1.5 mM KH_2_PO_4_), harvested with trypsin-EDTA digestion, and then centrifuged at 2, 000 rpm for 5 minutes. The cell pellets were resuspended with PBS, and the cytoplasmic and nuclear fractions were prepared by using NE-PER Nuclear and Cytoplasmic Extraction Reagents (Thermo Fisher, #78833), following the manufacturer’s instruction. GAPDH and Histone-H3 were used as the respective cytoplasmic and nuclear makers for monitoring fraction quality by Western blot.

### XBP1 mRNA splicing assay

Total RNAs from cultured cells were extracted with TRIzol reagent (Thermo Fisher, #15596026) according to the manufacturer’s instruction. The concentration of the extracted RNA was measured using a NanoDrop 1000 spectrophotometer (Thermo Fisher). Reverse transcription was performed using a FastKing RT Kit (with gDNase) (TIANGEN, #KR116-02) following the user guide. The XBP1 gene was amplified by PCR with the forward primer (F1) 5’-AAACAGAGTAGCAGCGCAGACTGC-3’ and the reverse primer (R1) 5’-GGATCTCTAAGACTAGAGGCTTGGTG-3’. To resolve the spliced forms (XBP1s) and unspliced (XBP1u) of XBP1, the PCR products were digested with the restriction enzyme *Pst* I-HF^®^ (NEB, #R3140L) and separated on the 1.5% agarose gel.

### Quantitative PCR

The cDNA was synthesized from the total cellular RNA by reverse transcription using the indicated primers. The relative quantitative PCR (qPCR) was performed with the Applied Biosystems^™^ SYBR^™^ Select Master Mix (Thermo Fisher, #4472913) according to the manufacturer’s recommendations. The PCR was assembled in a 20 μL reaction containing 0.2 μM gene specific primer, 10 μL SYBR^™^ Select Master Mix, and 2 μL cDNA template. The PCR parameter was set up as follows: 50°C for 2 min, 95°C for 2 min; 40 cycles of 95°C for 15 s, 60°C for 15 s, and 72°C for 60 s. To measure viral total RNA, a pair of internal primers in the ORF7 gene were used to amplify all sgmRNAs and gRNA. To measure gRNA and each viral sgmRNA, a pair primers in the corresponding ORF were used for the qPCR. The cellular GAPDH was quantified as the internal control to normalize the cDNA amounts. The 2^-ΔΔCT^ method was used to calculate the relative abundance of target genes compared to GAPDH. The primers used for qPCR are listed in S2 and S3 Tables.

### Lentivirus transduction

The lentiviral packaging system containing plasmids pWPXL (#12257), pMD2.G (#12259) and psPAX2 (#12260) was purchased from Addgene. The genes coding for XBP1s and ATF4 were cloned from MARC-145 cells into the plasmid pWPXL and expressed as c-Myc epitope fusion protein (XBP1s-myc and ATF4-myc). The recombinant viruses were rescued by co-transfecting the three plasmids into HEK-293FT cells. At 36 h post-transfection, the supernatants containing lentiviruses were harvested, filtered and concentrated. Titers of the lentiviruses were determined using a QuickTiter^™^ Lentivirus Titer Kit according to the manufacturer’s instruction (Cell Biolabs, #VPK-107). MARC-145 cells in 24-well plates were transduced with 1×10^6^ Lentiviral Particle (LP). At 36 h post-transduction, the cells were infected with PRRSV strain JXwn06 at an MOI of 0.1. The expression level of XBP1s and ATF4 was assessed by Western blotting with rabbit anti-myc polyclonal antibodies to measure the effect of overexpression of ATF4 and XBP1s on viral replication, and the total virus titer was assessed by end point dilution assay.

### RNA interference assay

For RNA interference, small interfering RNAs (siRNAs) was designed to target two different coding regions for each given gene. The sequences of siRNAs are listed in S1 Table. siRNAs were transfected with Lipofectamine^®^ RNAiMAX (Thermo Fisher, #13778150) according to the manufacturer’s instruction. Cell viability was assessed at 36 h post transfection with CellTiter 96^®^ AQueous One Solution Reagent (Promega, #G3582), and the knockdown effect was examined by Western blot analyses of the whole cell lysate with antibodies to ATF4, ATF6, GRP78 or XBP1s, and β-actin was used as a loading control. For transfection/infection assay, MARC-145 cells were infected with PRRSV strain JXwn06 at 36 h post transfection of siRNAs, and total viruses were collected at the indicated time points for titration, and total RNAs were extracted for analyzing the viral RNA abundance by relative qPCR.

### Western blot analysis

The amount of total cellular proteins was quantified by using Pierce^™^ BCA Protein Assay Kit (Thermo Fisher, #23225), and 20–30 μg of the whole lysate per sample was subject to western blot analysis. Briefly, the protein samples were separated by SDS-PAGE, transferred onto PVDF membranes, blocked with PBST (PBS with 0.05% Tween-20) containing 5% milk for 1.5 h, and then probed with appropriate primary antibodies at room temperature. For detection of phosphorylated proteins, bovine serum albumin (BSA) was used instead of milk. The membranes were then washed with PBST and incubated with appropriate HRP-conjugated secondary antibodies with dilution ratio 1:10, 000. The membranes were again washed and then developed with the Pierce ECL Western blot substrate (Thermo Fisher, #32209).

### Co-immunoprecipitation (Co-IP)

MARC-145 cells in six-well plates were infected with PRRSV strain JXwn06 at an MOI of 0.1. At 24 hpi, the cells were harvested and lysed in NP-40 lysis buffer (0.5% NP-40, 150 mM NaCl, 50 mM Tris-HCl pH 8.0) containing protease inhibitor (Sigma, #P8340). After clarifying by centrifugation at 12, 000 rpm for 20 min, the supernatants were precleared with protein A/G sepharose beads (Santa Cruz, #sc-2003) and then incubated with 5 μL mouse anti-nsp2 MAb (mouse isotype antibody as a negative controls) and 25 μL protein A&G sepharose beads overnight at 4°C with gentle rotation. The beads were washed five times with the NP-40 lysis buffer, and the proteins bounded to the beads were separated by SDS-PAGE, followed by western blot analysis.

### Confocal microscopy

MARC-145 cells grown to 60–70% confluence on coverslips in six-well plates were infected with strain JXwn06 at an MOI of 0.1. At indicated time points post infection, the cells were fixed with 3.7% paraformaldehyde for 10 min at room temperature, permeabilized with PBS containing 0.2% Triton X-100 and 2% bovine serum albumin (BSA) for 10 min, and then blocked with 2% BSA-PBS (2% bovine serum albumin in PBS) for 30 min. The cells were then incubated with primary antibodies as indicated in a humid chamber for 1 h at room temperature or overnight at 4°C. After being washed 3 times with PBS for 5 min each, the cells were incubated with appropriate second antibody (Alexa 488, 647 or 568-conjugated) for additional 1 h. Nuclear DNA was stained with 4’,6-diamidino-2-phenylindole (DAPI) (Thermo Fisher, #62248) for 10 min and then washed with PBS three times for 5 min each. The cells were imaged using a Nikon A1 confocal microscope.

### RNAscope *in situ* hybridization

To detect negative-stranded viral RNAs, *in situ* hybridization was employed by using the RNAscope^®^ Multiplex Fluorescent Detection Reagents v2 Kit (ACD, #323110). A total of 20 double-Z branched pairs targeting the regions of ORF6, ORF7 and 3’UTR of the PRRSV genome (nt position 14, 286–15, 273) as RNA probe (ACD, #510989) to detect negative-strand PRRSV RNAs. The hybridization procedure was performed according to the manufacturer’s instruction. Briefly, MARC-145 cells on Lab-Tek II Chamber Slides (Thermo Fisher, #154534) were infected with PRRSV at an MOI of 0.1. At the indicated time points post infection, the cells were fixed with 10% neutral buffered formalin (NBF), followed by dehydration and rehydration steps with suggested concentrations of ethanol and subsequent treatment by hydrogen peroxide (ACD) and protease III (ACD). The cells were then incubated with RNA probes for 2 h at 40°C in HybEZ^™^ Oven (ACD), followed by a cascade of signal amplification and a series of washing procedures. Hybridization signals were detected by TSA^®^ Plus Cyanine 3 (PerkinElmer, #NEL744E001KT). For triple staining, the cells were fixed again with 3.7% paraformaldehyde and then processed following the normal immunofluorescence assay procedure as described above.

### RNA immunoprecipitation (RIP)

RNA immunoprecipitation was carried out using the EZ-Magna RIP^™^ RNA-Binding Protein Immunoprecipitation Kit by following the manufacture’s protocol (Merck Millipore). Briefly, MARC-145 cells in 100 mm petri dish were infected with PRRSV strain JXwn06 at an MOI of 0.1 for 36 h and then washed twice with ice-cold PBS. The cells were then scraped off the plates, collected by centrifugation, and lysed by complete RIP Lysis Buffer with protease inhibitor cocktail (Sigma, #P8340) and RNase inhibitor (Thermo Fisher, #AM2694), followed by a step of centrifugation at 14, 000 rpm for 10 min at 4°C to remove the cell debris. To immunoprecipitate ATF4-RNA complexes, magnetic beads were mixed with rabbit anti-ATF4 polyclonal antibodies or normal rabbit IgG (isotype control), and then added to the clarified cell supernatants for incubation overnight at 4°C. After washing with cold RIP wash buffer six times, the beads-protein-RNA complexes were digested with proteinase K at 55°C for 30 min. Afterwards, the supernatants were collected on a magnetic separator and transferred to a new tube for RNA exaction with TRIzol reagent (Thermo Fisher, #15596026). The abundance of viral RNAs was analyzed by relative RT-qPCR targeting PRRSV 5’UTR or GAPDH (as a control).

### GST Pulldown assay

*E*. *coli* BL21 cells containing the plasmid pGST-ATF4 or pGEX-6P-1 vector were grown in 100 mL 2 x YT (yeast extract and tryptone) media at 37°C. Protein expression was induced for 3 h with Isopropyl β-D-1-thiogalactopyranoside (IPTG) at a concentration of 0.1 mM when the optical density at 600 nm reached 0.6. The cultures were pelleted at 8, 000 rpm for 5 min, resuspended in PBS supplemented with bacterial protease inhibitors, sonicated, and lysed for 30 min on ice with 1% Triton X-100. The lysates were cleared twice at 12, 000 rpm for 10 min at 4°C, and the supernatants were incubated with glutathione-Sepharose 4B beads at room temperature for 1 h, washed 3 times with PBS, and resuspended in 300 μL PBS.

To test the interaction with viral RNA *in vitro*, total RNA was extracted from PRRSV-infected MARC-145 cells using TRIzol reagent, and 300 μg were used for incubation with GST-ATF4 or GST beads in 300 μL NP40 Buffer (0.1% NP-40, 150 mM NaCl, 10 mM Tris-HCl, pH 7.4) with protease inhibitor cocktail (Sigma, #P8340) and RNase inhibitors (Thermo Fisher, #AM2694) overnight at 4°C. After washing six times with NP-40 buffer, the protein-RNA complexes were eluted from beads with 300 μL elution buffer (5 mM Tris-HCl, pH 7.5, 1 mM EDTA, pH 8.0, 0.05% sodium dodecyl sulfate), and the proteins were digested at 50°C for 0.5 h using proteinase K at a concentration of 0.28 mg/mL. Afterwards, the supernatants were collected by centrifugation at 12, 000 rpm for 2 min and transferred to a new tube where the abundance of viral RNA was analyzed by relative RT-qPCR targeting PRRSV 5’UTR or GAPDH as a control.

### Virus infection and titration

Following treatment with siRNAs or lentivirus transduction, MARC-145 cells were infected with PRRSV strain JXwn06. After incubation at 37°C for 1 h, unbound viruses were washed off with serum-free DMEM three times, and the cell cultures were supplemented with maintenance medium containing 2% FBS. At indicated time points post infection, the whole cell culture was collected, and the virus was titrated by the end point dilution assay.

### Statistical analysis

All the graphs and relevant statistical tests used in the work were created by GraphPad Prism version 6.00 (La Jolla, CA, USA). Statistical significance between two groups was analyzed by two-tailed unpaired Student’s *t*-test or one-way ANOVA, and asterisks indicate the statistical significance: NS, no significance; *, *P* < 0.05; **, *P* < 0.01; ***, *P* < 0.001. Error bars indicate means ± standard deviations (SD).

## Supporting information

S1 Fig Screening of PRRSV nonstructural proteins (nsps) for GRP78-degradation activity. MARC-145 cells on coverslips in six-well plates were transfected to individually express the indicated proteins tagged with an HA epitope at their N termini. At 24 h post transfection, the cells were fixed and stained with antibodies against GRP78 and the HA tag. Data information: Representative images were obtained by Nikon A1 confocal microscope. Oil objective: 100 X; zoom in 1 X.
(TIF)

S2 Fig Screening of PRRSV structural proteins for GRP78-degradation activity. (**A**) MARC-145 cells on coverslips in six-well plates were transfected to individually express the indicated proteins tagged with an HA epitope at their C termini. At 24 h post transfection, the cells were fixed and stained with antibodies against GRP78 and the HA tag. (**B**) Percentage of cells expressing structural proteins that showed increased (left panel) or decreased (right panel) fluorescence intensity of GRP78 as compared to non-transfected cells, which was measured by image J software (*n* = 50). Data information: Statistical analysis was performed by two-tailed Student’s *t*-test and error bars indicate means ± standard deviations (SD). Asterisks (*) indicate the statistical significance: *, *P* < 0.05; **, *P* < 0.01; NS, no significance. Representative images were obtained by Nikon A1 confocal microscope. Oil objective: 100 X; zoom in 1 X.
(TIF)

S3 Fig Effects of PRRSV infection on ATF4 nuclear translocation and downstream target gene expression. (**A**) MARC-145 cells were infected with PRRSV strain JXwn06 at an MOI of 0.1, and at 24 hpi, they were treated or untreated with TG (200 nM) for 0.5 h, fixed, and immunostained with antibodies against ATF4 and nsp2. Data information: Representative images were obtained by Nikon A1 confocal microscope. Oil objective: 100 X; zoom in 2 X. (**B**) MARC-145 cells (left panel) or PAMs (right panel) were either mock infected, infected with PRRSV strain JXwn06 at an MOI of 0.1, or treated with TG. At 24 hpi, cell lysates were prepared and analyzed by Western blotting with antibodies against GADD34, ATF4, actin, or the viral nucleocapsid. (**C**) The cells were collected for RT-qPCR with primers specific for ASNS mRNA, normalized against mRNA from the house-keeping gene GAPDH, and then compared to mock group. TG treated-cells were used as positive control.
(TIF)

S4 Fig PRRSV hijacks ATF4 to viral RTC in infected PAMs. Primary porcine pulmonary alveolar macrophages (PAMs) were grown on coverslips in six-well plates, and either mock-infected or infected with PRRSV strain JXwn06 at an MOI of 0.1. At 16 hpi, control groups were treated with TG or DMSO for 30 min, and then the cells were fixed and stained with antibodies against ATF4, nsp2 and nsp9. Data information: Representative images were obtained by Nikon A1 confocal microscope. Oil objective: 100 X; zoom in 2 X (large field) or 4 X (small field).
(TIF)

S5 Fig Hijacking ATF4 is a general property of PRRSV. MARC-145 cells were infected with the classical PRRSV strain HB1/3.9 and the NADC30-like PRRSV strain CHsx1401 at an MOI of 0.1. At 24 hpi, the cells were fixed and stained antibodies against ATF4 and nsp9. Data information: Representative images were obtained by Nikon A1 confocal microscope. Oil objective: 100 X; zoom in 1 X.
(TIF)

S6 Fig EAV and other RNA viruses do not retain ATF4 in the cytoplasm. Vero, BHK-21 and ST cells were seeded on coverslips within six-well plates, either mock-infected or infected with indicated viruses. At 24 hpi, the cells were stained with antibodies against ATF4 or the indicated viral component. (**A**) Localization analysis of ATF4 in Vero cells infected with PEDV (MOI = 0.05). (**B**) Localization analysis of ATF4 in BHK-21 cells infected with EAV (MOI = 0.05) and EMCV (MOI = 0.01). EAV was detected with mouse antibodies specific for dsRNA. (**C**) Localization analysis of ATF4 in ST cells infected with CSFV (MOI = 0.05). Data information: Representative images were obtained by Nikon A1 confocal microscope. Oil objective: 100 X; zoom in 1.5 X.
(TIF)

S7 Fig Screening of PRRSV nonstructural proteins for ATF4 cytoplasmic-retention activity. (**A**) Organization of the PRRSV genome. (**B**) MARC-145 cells on coverslips within six-well plates were transfected to express the indicated individual viral proteins tagged with an HA epitope at their N-termini. At 24 h post transfection, the cells were treated with TG (200 nM) for 0.5 h, and then they were fixed and stained with antibodies against ATF4 and the HA tag. Data information: Representative images were obtained by Nikon A1 confocal microscope. Oil objective: 100 X; zoom in 1 X.
(TIF)

S8 Fig Effect of ATF4 knockdown on the accumulation of individual PRRSV RNA species. MARC-145 cells were transfected with siRNAs targeting ATF4 (siATF4) or scrambled siRNA (siNC). At 36 h post transfection, the cells were infected with PRRSV strain JXwn06 at an MOI of 1. At the indicated times after infection, the abundance of individual positive- and negative-strand viral RNA species in the knock-down cells relative to the scrambled siNC control cells was measured by RT-qPCR and normalized relative to GAPDH mRNA. The specificity of sgmRNA2-7 qPCR primers was examined by electrophoresis in a 2–3% agarose gel following RT-PCR. (**A**) Analysis of the abundance of the positive strand of gRNA and sg mRNAs2-7. (**B**) Analysis of the abundance of the negative strand of gRNA and sg mRNAs2-7.
(TIF)

S1 Table Sequences for RNA interference. (DOCX)

S2 Table Primers sequence for qPCR. F: forward primer; R: reverse primer.
(DOCX)

S3 Table Primer sets for quantification of sgmRNAs and gRNA. gRNA: genomic RNA; sgmRNA: subgnomic mRNA.
(DOCX)
