# Supplementary figures and images for: Reprogramming the unfolded protein response for replication by porcine reproductive and respiratory syndrome virus
Source: PLoS Pathog. 2019 Nov 18;15(11):e1008169. doi: 10.1371/journal.ppat.1008169 (PMC6932825; doi:10.1371/journal.ppat.1008169)

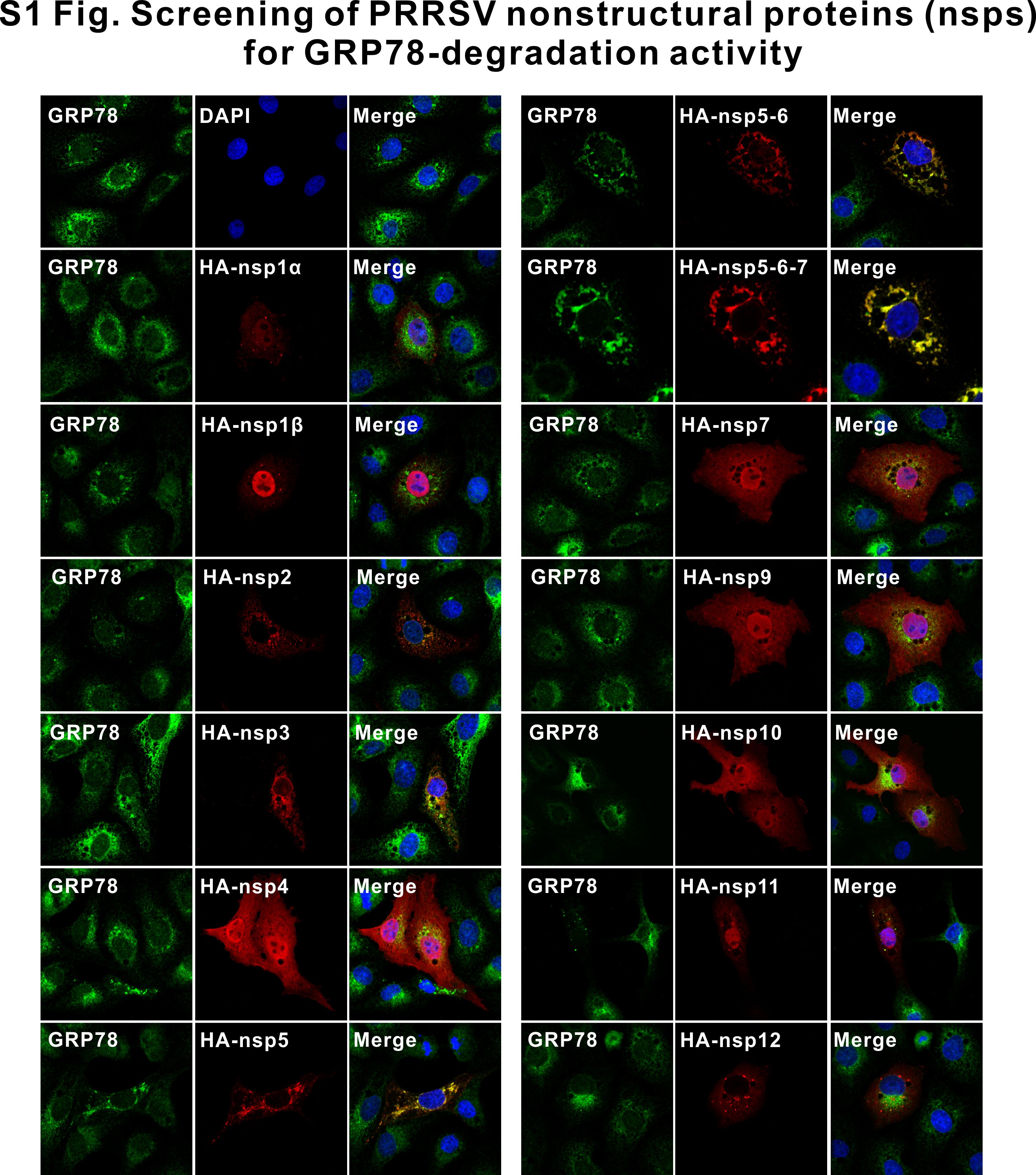

Supplement: S1 Fig — MARC-145 cells on coverslips in six-well plates were transfected to individually express the indicated proteins tagged with an HA epitope at their N termini. At 24 h post transfection, the cells were fixed and stained with antibodies against GRP78 and the HA tag. Data information: Representative images were obtained by Nikon A1 confocal microscope. Oil objective: 100 X; zoom in 1 X. (TIF) [file ppat.1008169.s001.tif]

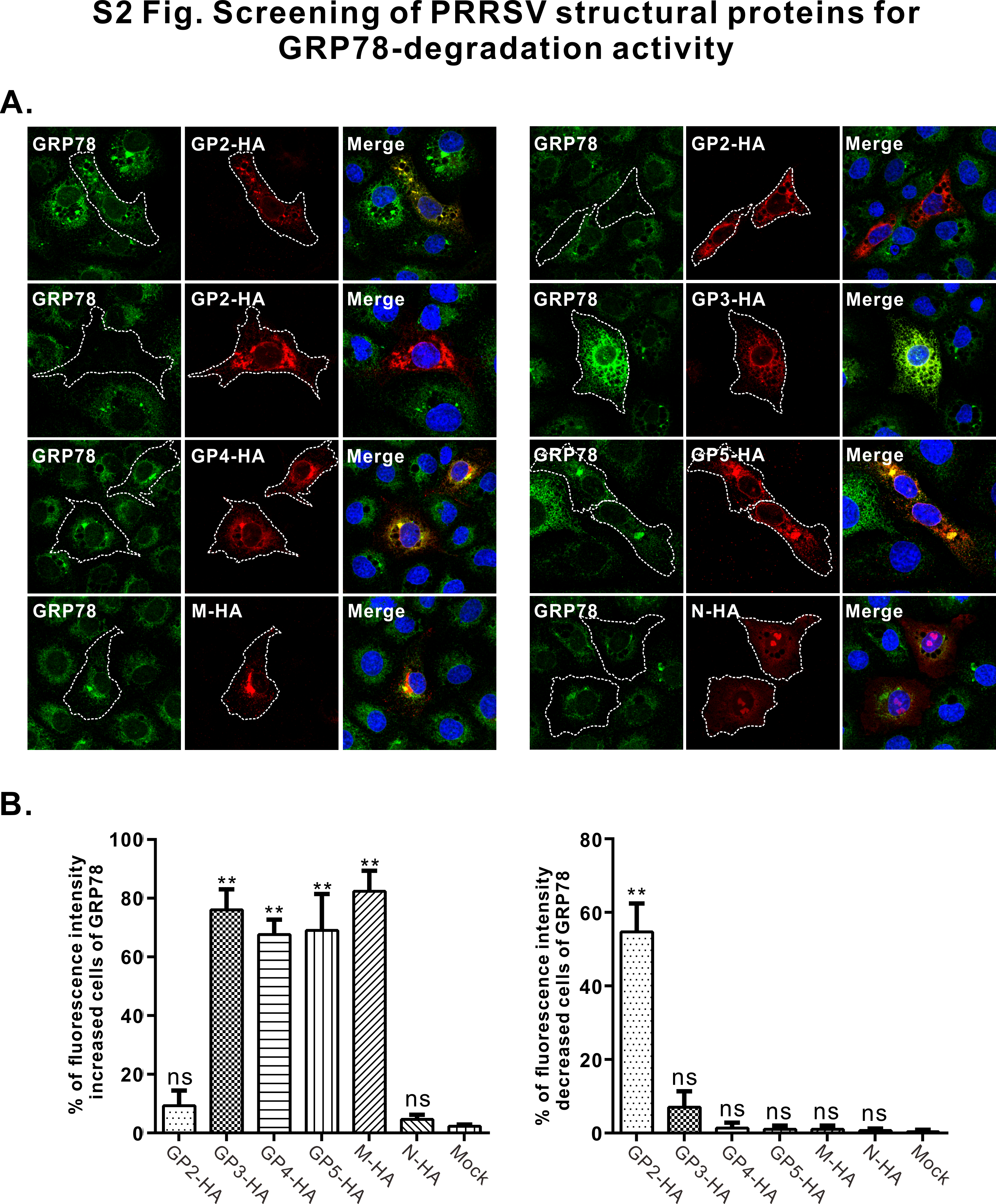

Supplement: S2 Fig — (A) MARC-145 cells on coverslips in six-well plates were transfected to individually express the indicated proteins tagged with an HA epitope at their C termini. At 24 h post transfection, the cells were fixed and stained with antibodies against GRP78 and the HA tag. (B) Percentage of cells expressing structural proteins that showed increased (left panel) or decreased (right panel) fluorescence intensity of GRP78 as compared to non-transfected cells, which was measured by image J software (n = 50). Data information: Statistical analysis was performed by two-tailed Student’s t-test and error bars indicate means ± standard deviations (SD). Asterisks (*) indicate the statistical significance: *, P < 0.05; **, P < 0.01; NS, no significance. Representative images were obtained by Nikon A1 confocal microscope. Oil objective: 100 X; zoom in 1 X. (TIF) [file ppat.1008169.s002.tif]

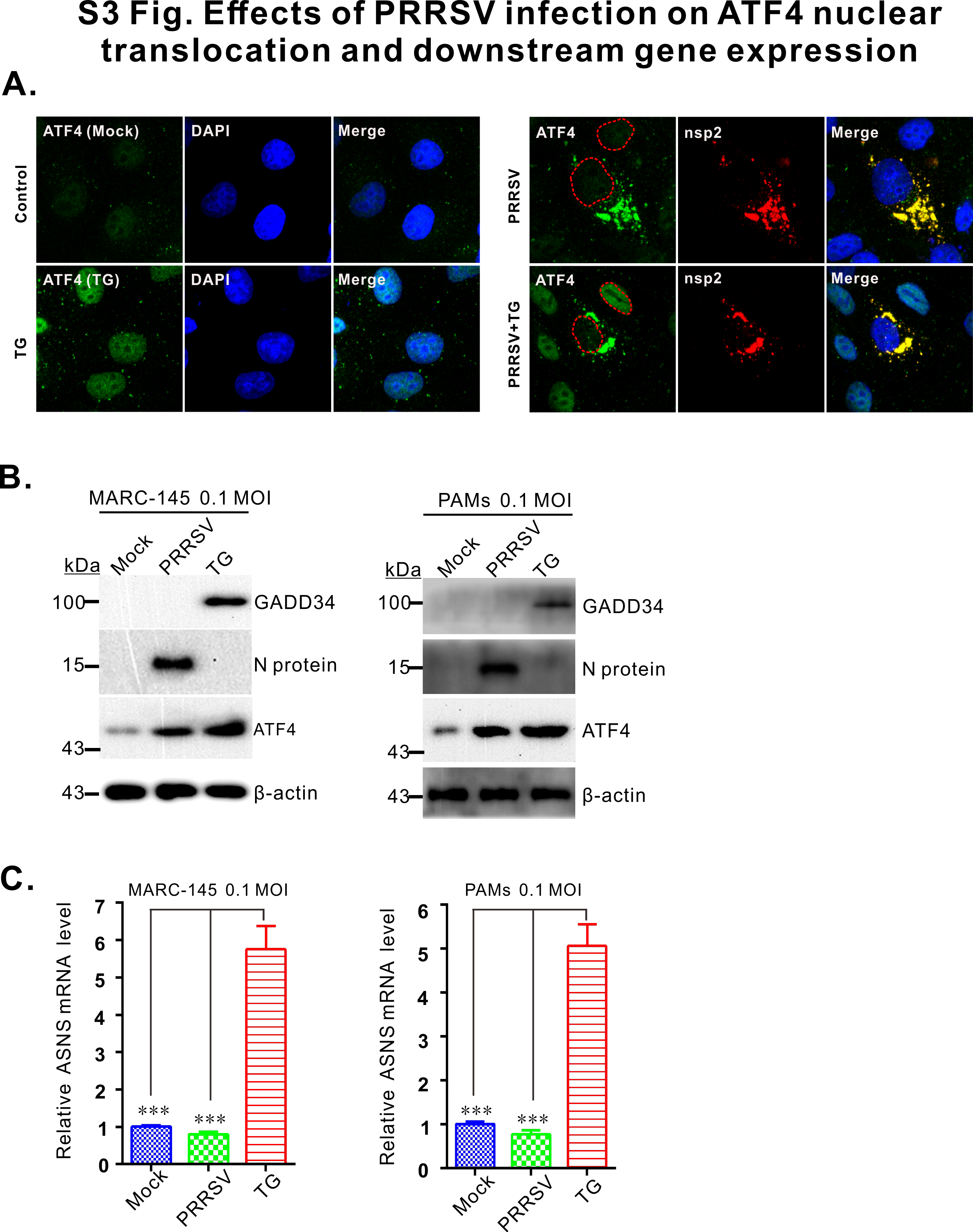

Supplement: S3 Fig — (A) MARC-145 cells were infected with PRRSV strain JXwn06 at an MOI of 0.1, and at 24 hpi, they were treated or untreated with TG (200 nM) for 0.5 h, fixed, and immunostained with antibodies against ATF4 and nsp2. Data information: Representative images were obtained by Nikon A1 confocal microscope. Oil objective: 100 X; zoom in 2 X. (B) MARC-145 cells (left panel) or PAMs (right panel) were either mock infected, infected with PRRSV strain JXwn06 at an MOI of 0.1, or treated with TG. At 24 hpi, cell lysates were prepared and analyzed by Western blotting with antibodies against GADD34, ATF4, actin, or the viral nucleocapsid. (C) The cells were collected for RT-qPCR with primers specific for ASNS mRNA, normalized against mRNA from the house-keeping gene GAPDH, and then compared to mock group. TG treated-cells were used as positive control. (TIF) [file ppat.1008169.s003.tif]

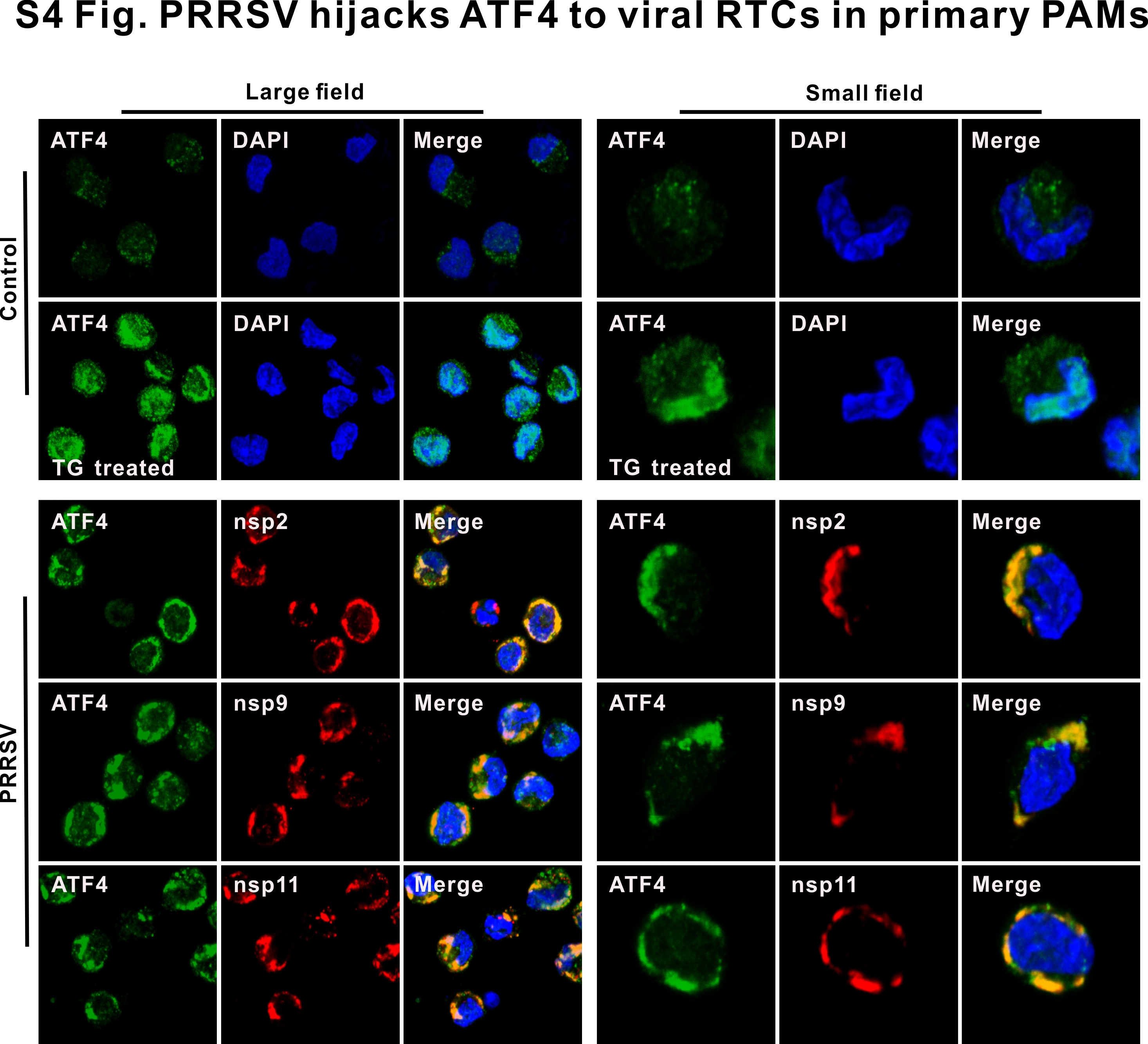

Supplement: S4 Fig — Primary porcine pulmonary alveolar macrophages (PAMs) were grown on coverslips in six-well plates, and either mock-infected or infected with PRRSV strain JXwn06 at an MOI of 0.1. At 16 hpi, control groups were treated with TG or DMSO for 30 min, and then the cells were fixed and stained with antibodies against ATF4, nsp2 and nsp9. Data information: Representative images were obtained by Nikon A1 confocal microscope. Oil objective: 100 X; zoom in 2 X (large field) or 4 X (small field). (TIF) [file ppat.1008169.s004.tif]

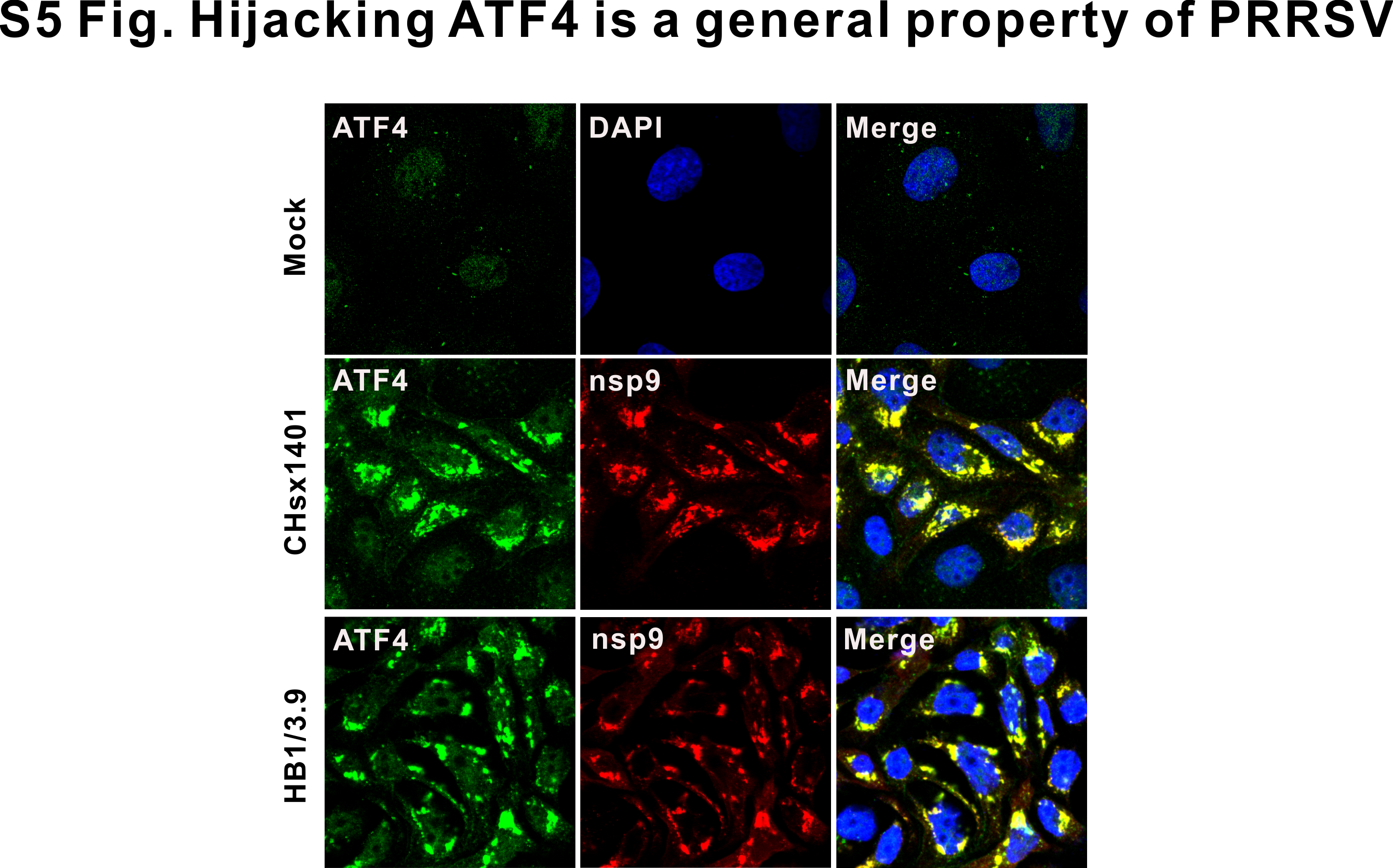

Supplement: S5 Fig — MARC-145 cells were infected with the classical PRRSV strain HB1/3.9 and the NADC30-like PRRSV strain CHsx1401 at an MOI of 0.1. At 24 hpi, the cells were fixed and stained antibodies against ATF4 and nsp9. Data information: Representative images were obtained by Nikon A1 confocal microscope. Oil objective: 100 X; zoom in 1 X. (TIF) [file ppat.1008169.s005.tif]

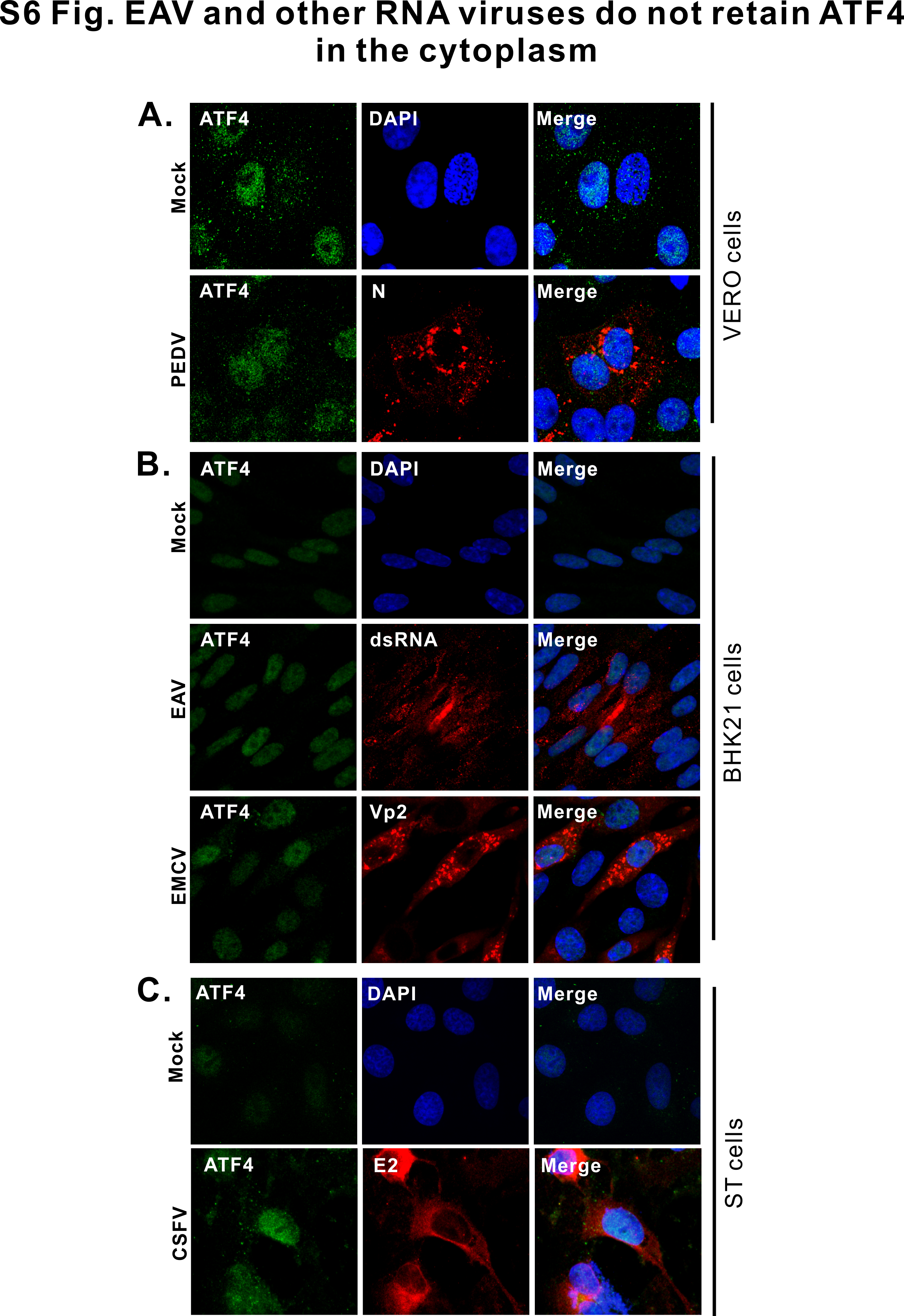

Supplement: S6 Fig — Vero, BHK-21 and ST cells were seeded on coverslips within six-well plates, either mock-infected or infected with indicated viruses. At 24 hpi, the cells were stained with antibodies against ATF4 or the indicated viral component. (A) Localization analysis of ATF4 in Vero cells infected with PEDV (MOI = 0.05). (B) Localization analysis of ATF4 in BHK-21 cells infected with EAV (MOI = 0.05) and EMCV (MOI = 0.01). EAV was detected with mouse antibodies specific for dsRNA. (C) Localization analysis of ATF4 in ST cells infected with CSFV (MOI = 0.05). Data information: Representative images were obtained by Nikon A1 confocal microscope. Oil objective: 100 X; zoom in 1.5 X. (TIF) [file ppat.1008169.s006.tif]

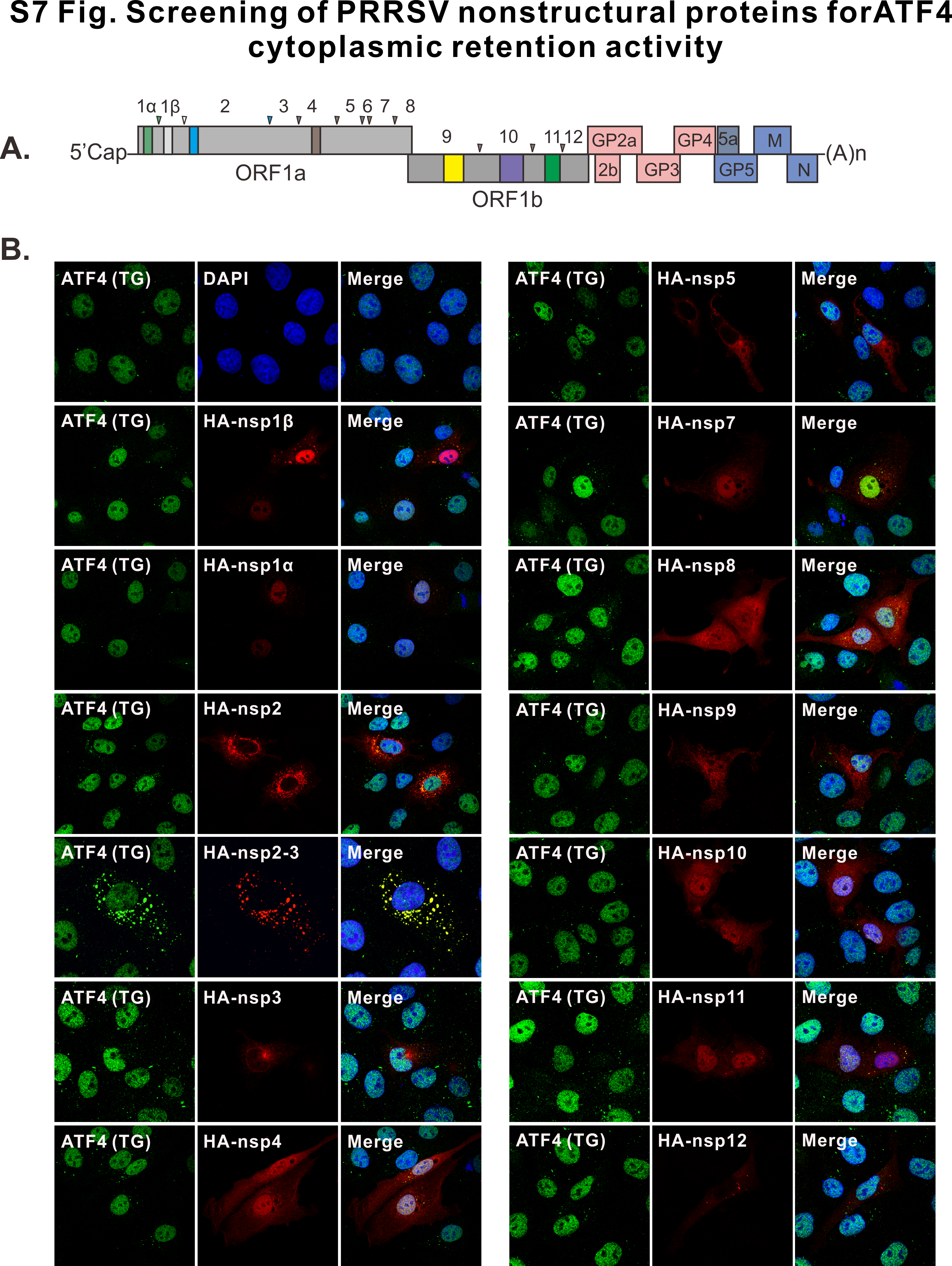

Supplement: S7 Fig — (A) Organization of the PRRSV genome. (B) MARC-145 cells on coverslips within six-well plates were transfected to express the indicated individual viral proteins tagged with an HA epitope at their N-termini. At 24 h post transfection, the cells were treated with TG (200 nM) for 0.5 h, and then they were fixed and stained with antibodies against ATF4 and the HA tag. Data information: Representative images were obtained by Nikon A1 confocal microscope. Oil objective: 100 X; zoom in 1 X. (TIF) [file ppat.1008169.s007.tif]

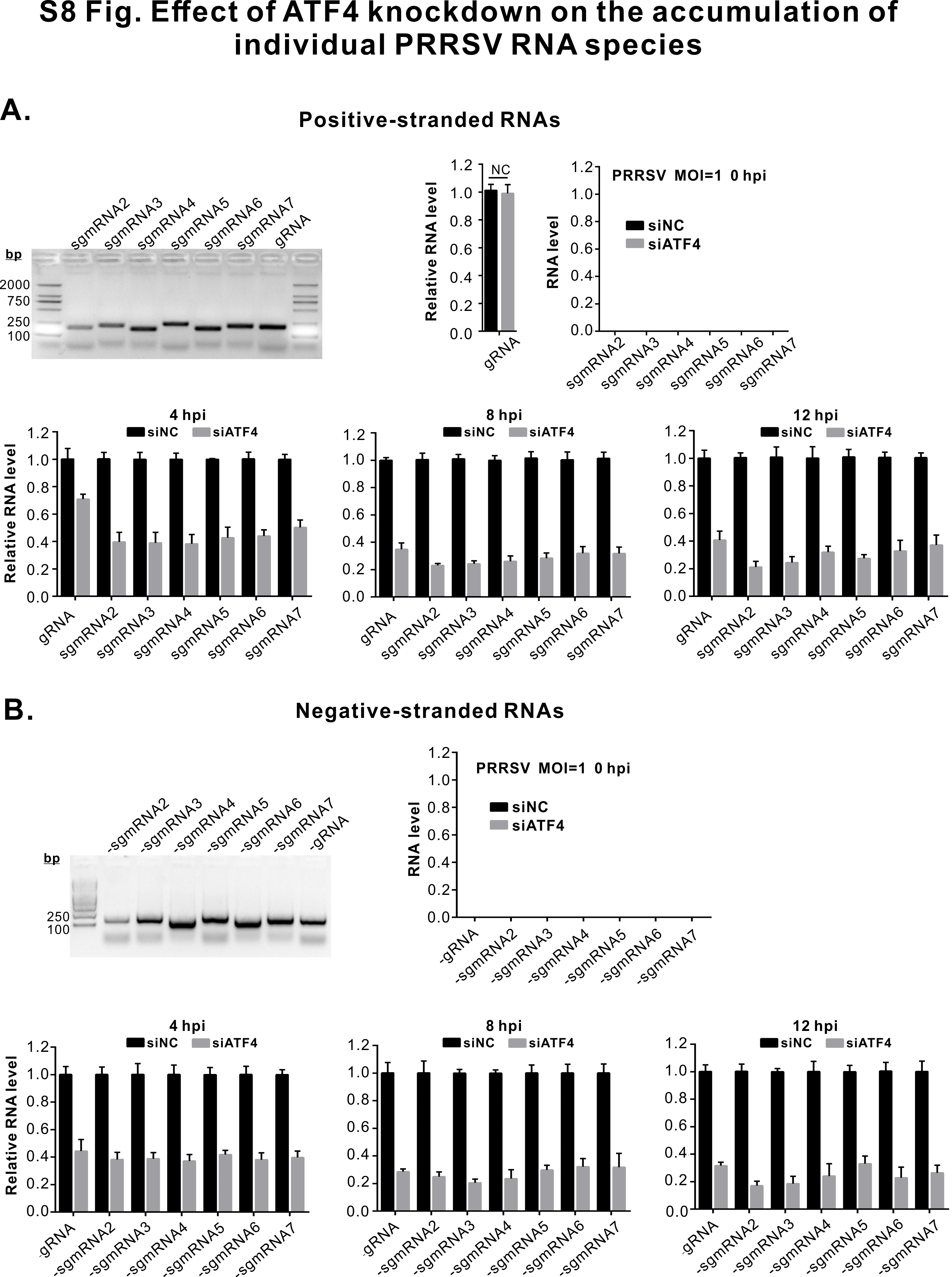

Supplement: S8 Fig — MARC-145 cells were transfected with siRNAs targeting ATF4 (siATF4) or scrambled siRNA (siNC). At 36 h post transfection, the cells were infected with PRRSV strain JXwn06 at an MOI of 1. At the indicated times after infection, the abundance of individual positive- and negative-strand viral RNA species in the knock-down cells relative to the scrambled siNC control cells was measured by RT-qPCR and normalized relative to GAPDH mRNA. The specificity of sgmRNA2-7 qPCR primers was examined by electrophoresis in a 2–3% agarose gel following RT-PCR. (A) Analysis of the abundance of the positive strand of gRNA and sg mRNAs2-7. (B) Analysis of the abundance of the negative strand of gRNA and sg mRNAs2-7. (TIF) [file ppat.1008169.s008.tif]
