# Supplementary material for: Reprogramming the unfolded protein response for replication by porcine reproductive and respiratory syndrome virus
Source: PLoS Pathog. 2019 Nov 18;15(11):e1008169. doi: 10.1371/journal.ppat.1008169 (PMC6932825; doi:10.1371/journal.ppat.1008169)
Supplement: S1 Table — (DOCX) [file ppat.1008169.s009.docx]

| siRNA name | sequence (5’-3’) |
| --- | --- |
| siRNA-ATF4 (1) | GCCUUCUACGGGACAGAUUdTdT |
| siRNA-ATF4 (2) | GCCUUCUACGGGACAGAUUdTdT |
| siRNA-XBP1 (1) | CCCAGUCAUGUUCUUCAAAdTdT |
| siRNA-XBP1 (2) | UGGGCAUUCUGGACAACUUdTdT |
| siRNA-ATF6 (1) | CCAGCCUCCUCAAGUUAUUdTdT |
| siRNA-ATF6 (2) | GCACCCAAGACUCAAGCAAdTdT |
| siRNA-GRP78 | GCAACUGGUUAAAGAGUUCdTdT |
| siRNA-NC | UUCUCCGAACGUGUCACGUdTdT |

**S1 Table. Sequences for RNA interference.**
