# Supplementary material for: Reprogramming the unfolded protein response for replication by porcine reproductive and respiratory syndrome virus
Source: PLoS Pathog. 2019 Nov 18;15(11):e1008169. doi: 10.1371/journal.ppat.1008169 (PMC6932825; doi:10.1371/journal.ppat.1008169)
Supplement: S2 Table — F: forward primer; R: reverse primer. (DOCX) [file ppat.1008169.s010.docx]

**S2 Table. Primers sequence for qPCR.**

| Primer name | Sequence (5’-3’) |
| --- | --- |
| GRP78-monkey-F | AGAAACTTCGGCGTGAGGTG |
| GRP78-monkey-R | GAGTCGAGCCACCAACAAGA |
| GRP78-sus-F | AGAAACTCCGGCGCGAGGTA |
| GRP78-sus-R | GAGTAGAGCCACCAACAAGA |
| 5’UTR-PRRSV-F | ATGACGTATAGGTGTTGGCTC |
| 5’UTR-PRRSV-R | GGTTAAAGGGGTGGAGAGACCG |
| GAPDH-monkey/sus-F | TGATGACATCAAGAAGGTGGTGAAG |
| GAPDH-monkey-R | TCCTTGGAGGCCATGTGGGCCAT |
| GAPDH-sus-R | TCCTTGGAGGCCATGTGGACCAT |

F: forward primer; R: reverse primer
