# Supplementary material for: Reprogramming the unfolded protein response for replication by porcine reproductive and respiratory syndrome virus
Source: PLoS Pathog. 2019 Nov 18;15(11):e1008169. doi: 10.1371/journal.ppat.1008169 (PMC6932825; doi:10.1371/journal.ppat.1008169)
Supplement: S3 Table — gRNA: genomic RNA; sgmRNA: subgnomic mRNA. (DOCX) [file ppat.1008169.s011.docx]

| Primer name | Sequence (5’-3’) |
| --- | --- |
| gRNA-F | GTCTCTCCACCCCTTTAACC |
| gRNA-R | AATGCACGTGGCAACGTCCAC |
| sgmRNA2-F | CCCTTTAACC ATGAAATGGGGT |
| sgmRNA2-R | GGAGCAAACCAGTCTGATGC |
| sgmRNA3-F | CCCTTTAACC ATGGCTAATAGC |
| sgmRNA3-R | TTCAAGGATCTCAGCGGCTGC |
| sgmRNA4-F | CCCTTTAACC ATGGCTGCGTC |
| sgmRNA4-R | CCATGCCTAAGGCAGCTGATG |
| sgmRNA5-F | CCCTTTAACC ATGTTGGGGAAG |
| sgmRNA5-R | GGAAACAATGTGAGTCAACAC |
| sgmRNA6-F | CCCTTTAACC ATGGGGTCGTC |
| sgmRNA6-R | GAAGGTAAAAGCACAATTCAG |
| sgmRNA7-F | CCCTTTAACC ATGCCAAATAAC |
| sgmRNA7-R | GGTAAAGTGATGCCTGACGTC |

**S3 Table. Primer sets for quantification of sgmRNAs and gRNA.**

gRNA: genomic RNA; sgmRNA: subgnomic mRNA.
